# Genome sequencing reveals genes under selection for olfactory transduction in highland sheep

**DOI:** 10.1007/s00438-026-02477-1

**Published:** 2026-07-14

**Authors:** Anees Ahmad, Muhammad Ibrahim, Muhammad Yaqoob, Muhammad Fahim, Sher Hayat Khan, Israr Ud Din, Majid Khan, Sohail Ahmad

**Affiliations:** 1 Institute of Biotechnology and Genetic Engineering, The University of Agriculture, Peshawar, Pakistan; 2 Biocomputation Research Group, Centre for Computer Science and Informatics Research, University of Hertfordshire, Hatfield, UK; 3 Centre for Omic Sciences, Islamia College Peshawar, Peshawar, Pakistan

**Keywords:** Sheep adaptation, Selection signature, Sense of smell, Reproductive performance, Olfactory receptors, Whole genome sequencing

## Abstract

Kutta is an indigenous sheep breed found in the mountainous region of Swat, Pakistan. In this study we evaluated the performance of Kutta sheep within their natural habitat and identified genomic regions associated with environmental adaptability using whole genome sequencing. Flocks are mostly managed under a transhumant production system with year-round breeding. Kutta sheep demonstrated higher lambing efficiency under the available resources. Although the body size of rams and ewes was comparable, significant variation was observed among different age groups. Whole genome sequencing identified 15.46 million variants relative to Oar_v1.0 reference genome assembly. Exonic variants accounted for 2.3% of all protein coding gene variants and affected 6347 genes. These genes were primarily enriched in pathways related to G protein-coupled receptor signaling (700 genes) and olfactory transduction (500 genes). Selective sweep analysis detected 1930 genes within the regions of high homozygosity across the genome. Based on homozygosity patterns, biological relevance, and colocalization with known QTLs, 11 candidate genes were identified as putative selective signature. Five of these genes (OR4C6L, OR1086L, OR5AK2L, QR5T2L, OR9Q2) belonged to the olfactory receptor family. These olfactory receptor genes may contribute to high-altitude adaptation in Kutta sheep by enhancing their ability to locate food resources, recognize mates, and detect predators in challenging mountainous environments. Further screening of the OR4C6L sequence across 12 indigenous sheep breeds revealed marked differences between highland and lowland populations. Collectively, these findings provide comprehensive genomic insights into high-altitude adaptation of sheep and highlight the potential role of olfactory perception in survival and environmental fitness.

**Supplementary Information:**

The online version contains supplementary material available at 10.1007/s00438-026-02477-1.

## Introduction

The differences in sheep’s characteristics under different environmental conditions are mainly due to their adaptation to a particular habitat. The genomes of sheep breeds have known to contain selective signatures, which have widely been explored to identify genes and genomic areas subjected to artificial selection for production qualities and adaptability to various settings (Liu et al. 2022). Several studies have utilized a variety of statistical methodologies to identify selection signals in different sheep populations. These approaches consider the hierarchical structure of sheep populations, linkage disequilibrium data, and discriminate between recent and older selection signals (Fariello et al. 2014). Comparing selection signatures across breeds has shed light on the genetic basis of phenotypic characteristics, production attributes, and adaptability to novel habitats in sheep (Fariello et al. 2014; Zhang et al. 2021, 2023). Key genes under selection include those associated with tail fat deposition (*GLIS1*, *FGF9*, *PDGFD*) (Qi et al. 2024), milk production (*SUCNR1*, *PPARGC1A*) (Yuan et al. 2019), wool properties (*PTPN3*, *KRTAP20*, *PIK3R4*, *LHX2*), meat quality (*MEF2C*, *MECOM*, *MYF6*) (Mohamadipoor Saadatabadi et al. 2021) and reproduction (*UNC5C*, *BMPR1B*, *GLIS1*) (Lv et al. 2023).

Climate-mediated selection pressure impacts the genetic composition of breeds suited to various habitats and production techniques. Local sheep populations have also been separated into breeds via systematic breeding with well-defined aims, resulting in a vast diversity of breeds adapted to diverse conditions and with varied breeding goals (Ciani et al. 2020). Certain breeds have unique characteristics that allow them to survive in a variety of climates and situations. A study has found genes linked to GTPase regulator and peptide receptor functions for energy metabolism that are connected with adaptation to local climates (Lv et al. 2014). Bangladeshi indigenous sheep breeds have developed characteristics like loose coarse wool, fatty tissue reserves, and long legs that allow them to withstand extreme climatic circumstances such as heat stress, cyclones, and droughts (Rakib et al. 2022). Similarly, Awassi sheep have evolved loose coarse wool, and fatty tissue reserves to survive harsh heat and dry circumstances. Some sheep breeds have evolved large fat tails that act as an energy reserve in feed scarcity during winter (Berihulay et al. 2019). Several genomic loci have been identified under selection in Iranian sheep breeds which are linked to adaptation to hot climates in these breeds (Patiabadi et al. 2024). Understanding these adaptations is essential for breeding and conservation efforts to safeguard endangered breeds and develop sustainable sheep production systems (Zhang et al. 2022).

Kutta sheep are valued for their wool and capability to thrive in hilly areas. Kutta sheep are characterized by their black coats, thin tails, and small size with an average weight of 23.2 ± 0.34 kg. The breed is exclusively used to produce wool, which is subsequently used to manufacture the regionally famed “Lamsay & sharri” hand-woven cloth. The breed is extremely resistant to foot-and-mouth and enterotoxemia illnesses (Ahmad et al. 2009). Kutta sheep are critically threatened due to reckless crossbreeding with other exotic sheep breeds. In a previous survey, only 553 purebred individuals were found in the Swat area (Jalil et al. 2009), with 304 ewes and only 41 rams, which is less than the FAO limit (Rischkowsky and Pilling 2007).

The available studies regarding phenotype associations with genetics in indigenous livestock populations are mostly at individual gene level. One study investigated the association of the *DGAT1* gene with milk and meat production traits in cattle, buffalo, goats, and sheep, revealing significant correlations that can inform breeding programs (Khan et al. 2021). Similarly, another study has identified a novel single nucleotide polymorphism (SNP) in the *interleukin-6 (IL-6*) gene of Pakistani sheep, potentially linked to variations in immune response (Ali et al. 2011). Additionally, the association between SNPs in heat stress proteins *HSP70* and *HSP90* and the susceptibility of Pakistani sheep breeds to hemoparasitic infections was examined, providing insights into genetic factors influencing disease resistance (Sheraz et al. 2023). Variations in the *KRTAP6-3* gene and their association with wool characteristics in Pakistani sheep breeds and crossbreeds were explored, offering potential for genetic improvements in wool production (Ullah et al. 2020). We have recently identified genes under selection for body size, reproductive performance, and milk traits using whole genome data of two indigenous sheep breeds (Ibrahim et al. 2026).

Genetic variability at the whole genome level is essential to understand the number of genes affecting a particular physiological trait and gene-to-gene interactions. This study aimed to sequence the whole genome of Kutta sheep and conduct selective sweep analysis to identify genes under selection. The endangered status of the breed warrants study of its characteristics in association with genomic data as the breed may possess important characteristics which might help adapt livestock species to hilly areas and cold environments. Studying its genomic architecture and selection signature will not only aid in conservation efforts but also contribute to sustainable livestock management in challenging climatic conditions. Here we evaluate the performance and morphology of Kutta sheep in the Swat region. Then we analyze variants in the whole genome of Kutta sheep compared to the ovine reference genome. Gene ontology is performed for significant gene variants. Analysis of selection signatures identify potential genes under selection. Finally, we associate the reported roles of genes under selection with performance characteristics of Kutta sheep.

## Materials and methods

### Animal selection

Kutta sheep habituating the Kalam region in Swat Pakistan was selected for this research. Kutta is a small-size sheep breed mostly bred for wool and mutton production. The breed is well adapted to the cold and mountainous terrain of the Swat. Kalam valley is located in upper Swat at the bank of Swat River at an elevation of 6600 feet. The valley is surrounded by Matiltan in east, Utrar (Chitral) in the west, and Bahrain in south. The climate is characterized as humid subtropical with mean monthly temperature ranging from − 14 °C in January to 12 °C in July. Average precipitation ranges from 68 mm to 196 mm per month with heavy snowfall in winter (https://en.climate-data.org).

### Analysis of population structure

Kutta sheep flocks dwelling in Kalam and adjacent areas were visited in a survey. A total of 23 flocks were found in the area containing Kutta sheep. Interviews were conducted with the farmers. Data regarding flock size and structure, farming system, grazing and feeding practices, health management, breeding practice, reproductive performance, and sheep marketing were recorded on a questionnaire.

### Analysis of morphological features

Purebred Kutta sheep were identified based on their breeding history (as per the farmers´ records) and their morphological similarities with previously reported characteristics (Ahmad et al. 2009). Morphometric data were collected from a total of 130 Kutta sheep individuals containing 45 rams, 75 ewes, and 10 lambs. Sheep scattered in different flocks across the region were sampled to avoid genetic relatedness. Various morphometric traits were recorded including measurements (length, width, circumference etc.) of head, mouth, ears, neck, body, rump, and tail. Morphological features such as wool color, forehead type, nose type, horns type, and tail type were also recorded for individuals.

### Whole genome sequencing

Blood samples were collected from 10 Kutta ewes randomly selected from the Kalam region. DNA was extracted from these samples using NucleoSpin genomic DNA extraction kit (Machereay-Nagel GmbH & Co. KG, Duren, Germany, Catalog # 740951.250). DNA concertation and purity were checked using Qubit™ dsDNA kit (Invitrogen, USA, Catalog # Q32851). An equal amount of DNA from each sample was pooled in an Eppendorf tube for sequencing. Whole genome DNA sequencing was done through the Illumina NovaSeq platform at Beijing Genomic Institute (BGI). Paired-end sequencing (2 × 150 bp) was performed. Sequencing depth of the generated reads ranged from 20× to 25×. A total of 55.8 GB of raw data were generated, including 185.9 million raw reads, with a GC content of 44.1%. Of the total raw reads, 95.9% (Q20) and 89.9% (Q30) reads correctly mapped to the reference genome. The data were generated in FastQ format, which was further analyzed using the following bioinformatics pipeline.

### Bioinformatics analysis of whole genome data

The read quality for the fastq files were checked using FastQC v0.11.8. The results of this analysis were used as the basis for read quality filtering using the Trimmomatic tool v0.39, to delete index adapter sequences, low quality base calls (Q < 30) and index adapter sequences (Bolger et al. 2014). The filtered reads were aligned to the sheep reference genome (Oar_rambouillet_v1.0, RefSeq assembly accession No. GCF_002742125.1) using Burrows-Wheeler Aligner method v0.6 (Li and Durbin 2010) using default options. Picard tool v2.21.6 was used to remove the PCR duplicates from the reads.

Alignment is usually disturbed due to the presence of small InDels (insertions and deletions), to resolve this issue command-line tools of the Genome analysis toolkit (GATK v3.3.0) (McKenna et al. 2010). “RealignerTargetCreator” and “InDelRealigner” were used. The command-line in GATK, “BaseRecalibrator” was used to recalibrate base quality score (BQSR), to fix the changes in base quality score estimations due to sequencing artifacts. “HaplotypeCaller” in the GATK were used to call single nucleotide polymorphisms (SNPs) and insertions deletions (InDels). To remove the false variants that may have emerged in the raw variants call set, GATK’s tool “Variant Filtration” was used with default settings. The reported mutations were finally annotated using SnpEff v4.3t (Cingolani et al. 2012).

### Evaluating the total variants

Total number of variants in the Kutta genome compared to the reference genome were evaluated and counted in the output files of SnpEff. The numbers of SNPs and InDels were documented. The variants with high effects such as frameshift mutations, missense mutations, large coding insertions, and deletions, etc. were highlighted. Other variants including synonymous mutations, intron variants, upstream variants, downstream variants, 5ʹ and 3ʹ UTR variants etc. were extracted and tabulated form the SnpEff output file.

The genome contains different gene types; the protein coding genes are a major part of which. Other gene types include pseudogenes, miroRNA genes, nuclear RNA gene, ribosomal RNA genes, transfer RNA genes etc. Number of variants in each of these categories were identified and tabulated from the whole genome data of the Kutta sheep.

### Gene annotation

Genes effected with high impact variants such as frameshift mutations, large insertions, deletions in the coding region, and other non-synonymous mutations were identified and further evaluated for Gene Ontology. The Gene Ontology Resource (GO; http://geneontology.org) offers organized, computable information on the roles of genes and gene products. Database for Annotation, Visualization, and Integrated Discovery (DAVID) (Sherman et al. 2022), and Cytoscape software v3.8.2 (Shannon et al. 2003) were used to identify the GO terms of the selected genes and construct a gene ontology network.

### Selective sweep analysis

Pooled heterozygosity (ZH_P_) was calculated for genomic regions of Kutta sheep using the annotated file. The genome was split into windows of 600 kb size, and the ZH_P_ for each window was calculated using a Python script (https://github.com/yaqoobcs/selective-sweep-implementation) using the formula for H_P_ and ZH_P_ as mentioned in our previous study (Ibrahim et al. 2026). The selective sweep results were then plotted on a scatter plot using the matplotlib library in Python. H_P_ was initially calculated for different window sizes, from which 600 kb window size was selected showing peaks below ZH_P_ − 2.0. This threshold score was selected based on the presence of windows in the extreme lower tail of the genome-wide ZH_P_ distribution as suggested in previous studies (Rubin et al. 2010).

### Identification of candidate genes

The genomic regions below the threshold ZH_P_ score (–2.0) were searched in the NCBI genome annotation tool to identify the total number of protein-coding genes present in these regions. The total number of genes found in these regions were then screened for the number of variants and their homozygosity in the VCF file. The genes containing at least one non-synonymous SNP, having greater than 1% total number of variants (number of variants/total gene length), and greater than 90% homozygosity (hard selective sweep containing < 10% heterozygous variants) were selected as candidate genes under selective sweep. The gene list was further narrowed down by evaluating their functional annotation using DAVID online tool and by searching for their colocalization with previously known QTLs in the Animal QTL database (https://www.animalgenome.org/cgi-bin/QTLdb/OA/index). Nucleotide diversities were calculated for further validation of homozygosity fixation in these genes.

### Nucleotide diversity

Nucleotide diversity of the genes identified as the selective signature of the Kutta sheep was calculated for 5 kb sliding windows using the following formula:

$$\:\pi\:={x}_{i}{x}_{j}{\pi\:}_{ij}$$

where *π* is the nucleotide diversity, *x*_*i*_ is the frequency of reference allele (i.e. the same allele found in reference genome and the genome of Kutta sheep at a locus), *x*_*j*_ is the frequency of alternate allele (i.e. a different Allele found in Kutta sheep compared to the reference genome), and *π*_*ij*_ is the number of nucleotide differences per site. The nucleotide diversity was plotted on a line graph using the matplotlib library in Python.

### Extracting gene data

The genome wide variants of 12 indigenous sheep breeds (unpublished data) including seven lowland breeds and five highland breeds were merged using Python Numpy library. The highland sheep breeds are namely Tirahi, Kari, Kutta, Ghalji, and Kaghani from the mountainous regions of Southern and Northern Khyber Pakhtunkhwa. The lowland sheep breeds are Balkhi, Hashtnagri, Michni, Madakhlasht, Mazai, Waziri, and Damani from the central and southern Khyber Pakhtunkhwa. See our previous study (Ibrahim et al. 2023) for the details of sampling location of each breed. Whole genome sequencing was performed for each breed using the same protocol as mentioned above for Kutta sheep. After merging the VCF files of all the 12 sheep breeds, the region containing variant data of the OR4C6L gene was extracted and analyzed for variations among lowland and highland sheep breeds.

### Statistical analysis

Percentages, mean, SE, and ANOVA were calculated using SPSS version 23.0 (IBM-Corp 2015). The sum of variants in different categories of gene types was also calculated from the SnpEff output using SPSS software. The selective sweep and nucleotide diversity results were plotted in scatter plot and line graph, respectively, using the matplotlib library in Python. Diversity parameters i.e. Hardy Weinberg equilibrium (HWE), Shannon index, inbreeding estimate (F_IS_) were calculated using the genotype data in POPGENE software version 1.32. Tjiama’s D score was calculated using the nucleotide diversity data using the formula given by ref. (Korneliussen et al. 2013).

## Results

**Fig. 1:**
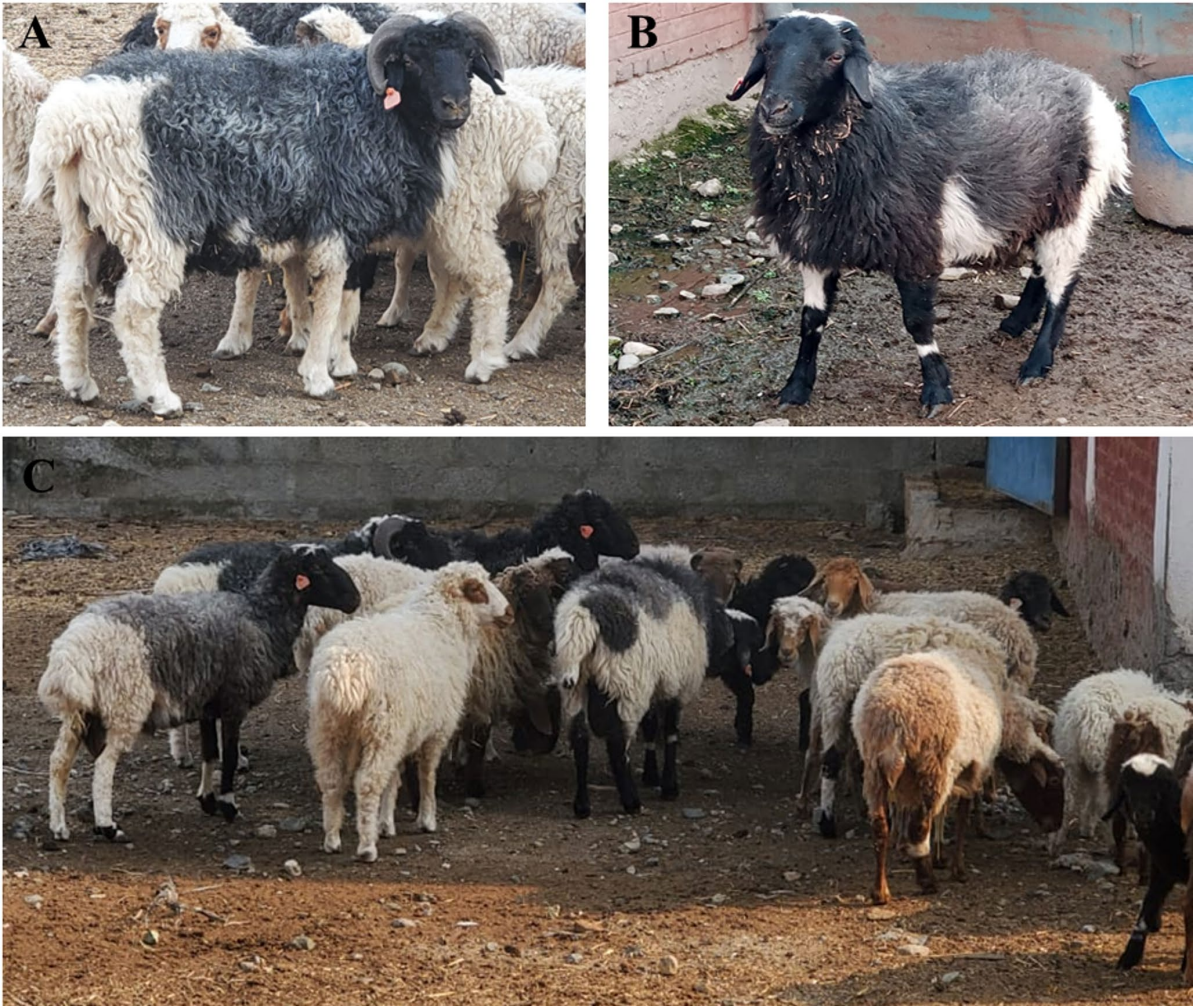
Representative images of Kutta sheep in their natural habitat. **A** Kutta ram **B** Kutta ewe **C** A flock containing Kutta specimen

A total of 23 flocks containing Kutta sheep were found in the Kalam valley. Total number of sheep recorded in these flocks was 676, of which only 230 (34%) belong to Kutta sheep breed, while the remaining were crossbred, usually descended from Rambouillet or Balkhi sheep rams crossed with Kutta ewes. However, 10 out of 23 flocks were reportedly bred with Kutta rams. The flock size ranged from 5 to 35 sheep per flock with an average size of 29.4 ± 3.5. Generally, a flock consists of breeding rams (1.6 ± 0.2), adult ewes (16.7 ± 1.9), and lambs (11.0 ± 1.5). Representative pictures of Kutta sheep ram, ewe, and flock are shown in Fig. 1.

### Husbandry and performance of Kutta sheep

Most of the Kutta sheep flocks (82.6%) are managed under the transhumant system, where the flocks are migrated to warm regions during the months of winter. Under the sedentary system, sheep are housed in a proper shelter to reduce the risk of cold stress and diseases protection. Adequate care was taken for sheep’s health and productivity. The flocks were given adequate access to clean water, feed supplements, and grazing (Supplementary Table 1).

The flocks’ breeding system was well-organized. Rams were used in rotation to avoid inbreeding. On average the flocks contained 1.6 rams/flock. During the breeding seasons, rams were also borrowed from neighboring flocks for rotation. Average age of the rams was 26.8 ± 1.3 months, ranging from 18 to 36 months. The breeding follows a continuous system in most of the flocks, ensuring that rams are always available for mating (Supplementary Table 1). The lambing season usually occurs in spring (March – May), which provides ideal conditions for lamb growth and survival. Average age at first lambing was 18.6 ± 0.3 months. Lambs are naturally weaned through self-weaning by the age of 3 months.

The flock commonly suffer from several diseases, including Foot-and-Mouth Disease (FMD), enterotoxemia, diarrhea, and injuries, with pleuropneumonia being the predominant disease and the main cause of mortality. The vaccines available include those for sheep pox and bluetongue administered at 3–6 months with annual booster doses during the warm season; FMD and pasteurellosis that are given yearly during outbreaks or stress periods; and brucellosis and toxoplasmosis, which is given initially at 3–6 months and a booster dose after 12 months before the first breeding season. De-worming using albendazole in March, ivermectin in July, and levamisole in October is also regular practice. These preventive health measures are important for controlling diseases, reducing mortality, and maintaining the overall productivity of the flock (Supplementary Table 1).

### Morphology of Kutta sheep

Body morphometry of different categories of Kutta sheep is presented in Supplementary Tables 2 and 3. The lambs are usually smaller than adult ewes and rams; however, no significant differences were observed in the body size of rams and ewes (Supplementary Table 2). Significant differences were observed in the body size of different age groups (Supplementary Table 3); however, measurements of some body parts such as belly depth, body height at withers, rump size, and tail length and diameter remained similar. The color of different body parts of Kutta sheep are mostly black or white, with some brown specimen (Supplementary Table 4).

Head morphology was flat in 92.3% individual. age group of one year and less had more individuals with bulging forehead (20%) compared to the age group of 1–3 years (6.7%) and above. The kids less than 1 year age were polled, while 13.3% of 1–3 years age sheep were horned. The percentage of horned individuals increased to 66.7% for sheep aged above 3 years. Horns were more common in rams (44.4%) than ewes (13.3%).

### Total variants in Kutta genome compared to reference

The genome of Kutta sheep showed a total number of 15.46 million variants compared to the reference genome (Supplementary Fig. 1). According to the data, variants related to protein-coding genes constitute the majority (94.5%) variants. The protein coding gene variants were further subdivided into genic variants, which are dominant (87.9%), and the intergenic variants (12.1%), which include upstream and downstream variants. Within the genic variants the highest proportion was observed in the introns (97.3%), while the density of exon variants was much less frequent (2.7% of the genic variants). Variants were also observed in different types of RNA genes and pseudogenes (Supplementary Table 5).

### High impact coding gene variants

The types and numbers of protein-coding gene exon variants in the genome of Kutta sheep are presented in Table 1. The 3′ UTR variants are the most frequent, followed by synonymous variants. Suggesting that most of the exon variants have no significant effect on the function. Other exon variants including frameshift, start and stop codon variants, insertions, deletions, and missense variants might have significant effects on the protein structure and function. The total number of such variants was 52,548, which accounted for 15.3% of the total exon variants, affecting 6347 genes. In addition to the high impact exon variants, some intron variants (splice region variants) may also have significant impact on protein structure and function. These intron variants were also found at lower frequency in the genome of Kutta sheep (Table 1).

**Table 1:** High impact exon and intron variants affecting the protein coding genes in the genome of Kutta sheep

Variant types	Number	Percentage
Frameshift	2089	0.57
Start lost	106	0.03
Stop gained	375	0.10
Stop lost	111	0.03
Conservative deletion	195	0.05
Conservative insertion	221	0.06
Disruptive deletion	418	0.11
Disruptive insertion	340	0.09
Missense	43,748	11.95
Initiator codon	9	0.00
Stop retained	48	0.01
Premature start codon gain	4,888	1.33
Synonymous	92,414	25.24
5 prime UTR	37,999	10.38
Non-coding transcript	67	0.02
3 prime UTR	160,993	43.97
Splice acceptor	464	0.13
Splice donor	510	0.14
Splice region	21,184	5.79

*UTR*  untranslated region

### Ontology of the mutated genes in Kutta sheep

**Fig. 2:**
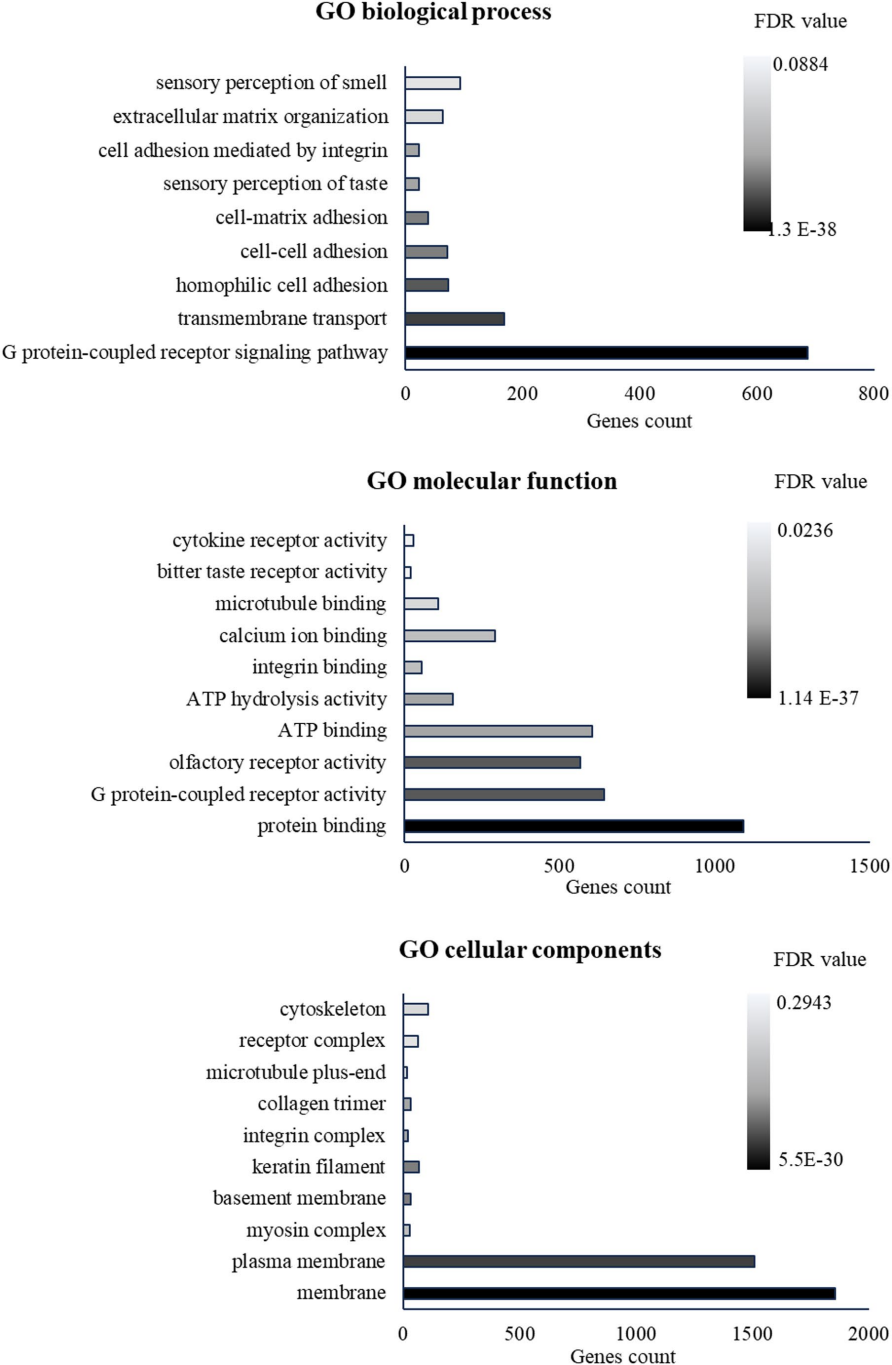
Number of mutated genes affecting biological processes, molecular functions, and cellular components. Top 10 GO pathways based on FDR values < 0.05 were selected the highest number of genes were involved in G-protein coupled receptor signaling pathway, protein binding, and membrane component

The genes showing high impact variants were checked for their functional annotation using DAVID online tool. This resource identified the enriched pathways associated with the genes list. The top 10 pathways regarding biological process, cellular component, and molecular function in which these genes are involved are shown in Fig. 2. The G protein-coupled receptor signaling pathway was the most effected biological process with the largest number of mutated genes (686) involved with highly significant FDR value. The second most significant biological process was transmembrane transport with 168 genes involved. The biological processes related to sensory perception, like odor and taste, also manifest enrichment with 94 genes involved.

The most significant enrichment in molecular functions includes protein binding and G protein-coupled receptor activity (Fig. 2), with large gene counts (1093 and 644, respectively), pointing to their critical role in signal transduction and molecular interactions. Supplementary functions include binding to ATP, interaction with calcium ions, and association with microtubules, suggesting roles in energy metabolism and structural regulation. Membrane and plasma membrane components were the most significant cellular components showing enrichment with the given gene list with 1855 and 1509 genes involved, respectively. These components are key in establishing cellular compartmentalization and signaling. Other enriched components include the cytoskeleton, integrin complex, and basement membrane, which represent structural and adhesion-related functions. Together, this analysis highlights key modifications in pathways and molecular mechanisms involved in signal transduction, adhesion, structural dynamics, and energy-related activities in Kutta sheep breed.

**Fig. 3:**
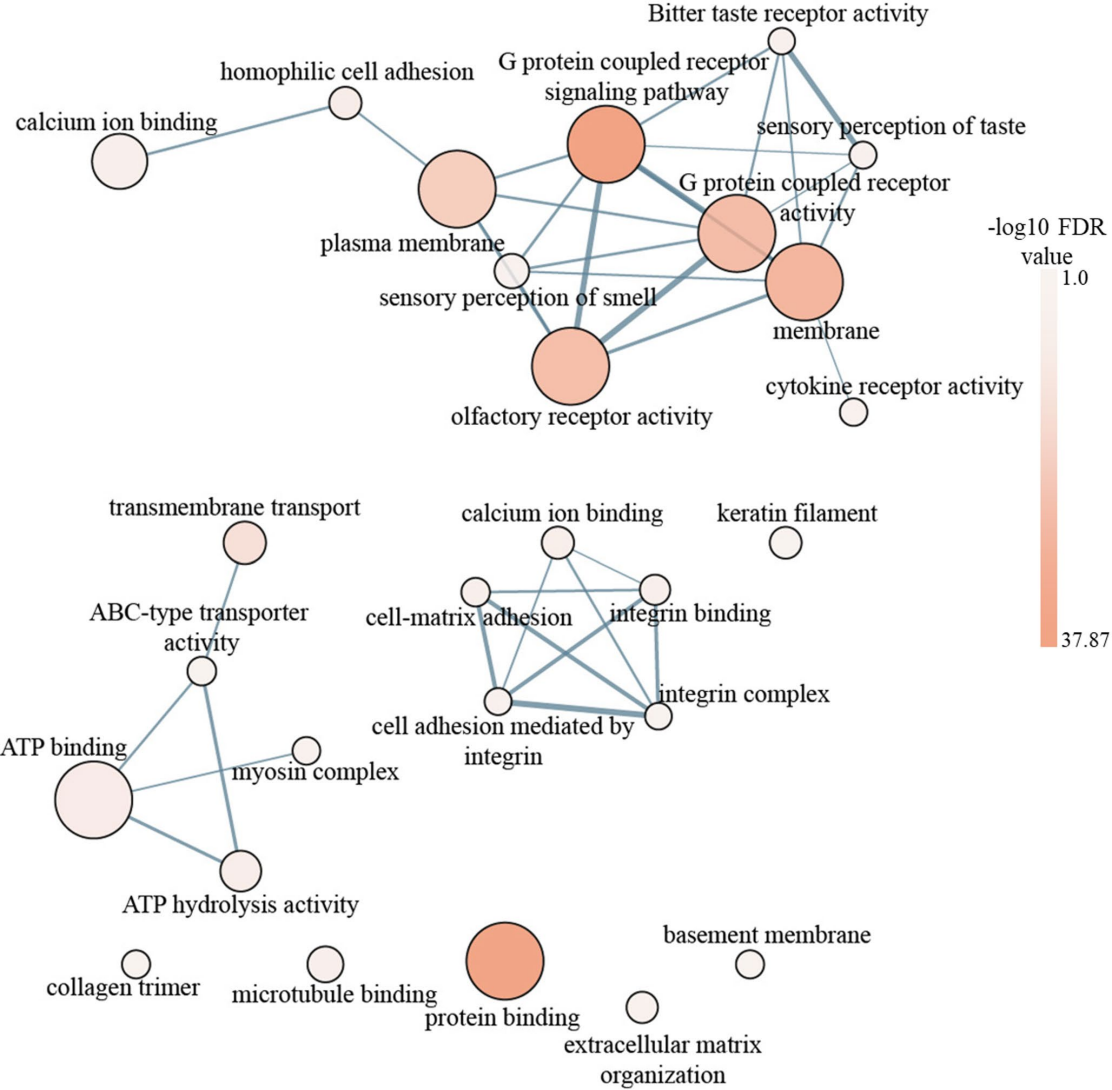
Gene Ontology network identifiying the number of genes involved in different biological process and their interrelationship. The size of circles indicates the number of genes. The color indicates FDR values. The thickness of the nodes between the circles indicates the number of common genes among different processes. The highest number of genes were involved in G-protein coupled receptor pathway, which share maximum number of genes with olfactory receptor activity. The lower segment of the network highlights adhesive and structural roles, specifically via cell-matrix adhesion and integrin signaling pathways. Key nodes, including integrin binding and cell-matrix adhesion, are notably abundant, indicating their essential function in facilitating cellular interactions with the extracellular matrix (ECM)

The visualization of Gene Ontology (GO) network depicts the interconnections among diverse biological processes, molecular functions, and cellular components linked to a specific gene or set of genes being examined (Fig. 3). The upper section of the network emphasizes signal transduction and sensory mechanisms, wherein G protein-coupled receptor (GPCR) activity is ascribed to a pivotal role. The activity of GPCRs and the associated signaling pathways exhibit a robust interconnection with processes including olfactory receptor function and the sensory experiences of smell and taste.

The prominence of major nodes, including the GPCR signaling pathway and olfactory receptor function, underscores their considerable enrichment and significance within the genome of Kutta sheep. Furthermore, the plasma membrane functions as an essential structural element, incorporating sensory activities such as the detection of bitter taste receptors and connecting external stimuli to intracellular reactions. This network further highlights the interrelated functions of membrane proteins in cytokine receptor engagement, olfactory processing, and sensory signaling, suggesting a wider role of these genes in mechanisms of environmental responsiveness.

### Genes involved in KEGG pathways

**Fig. 4:**
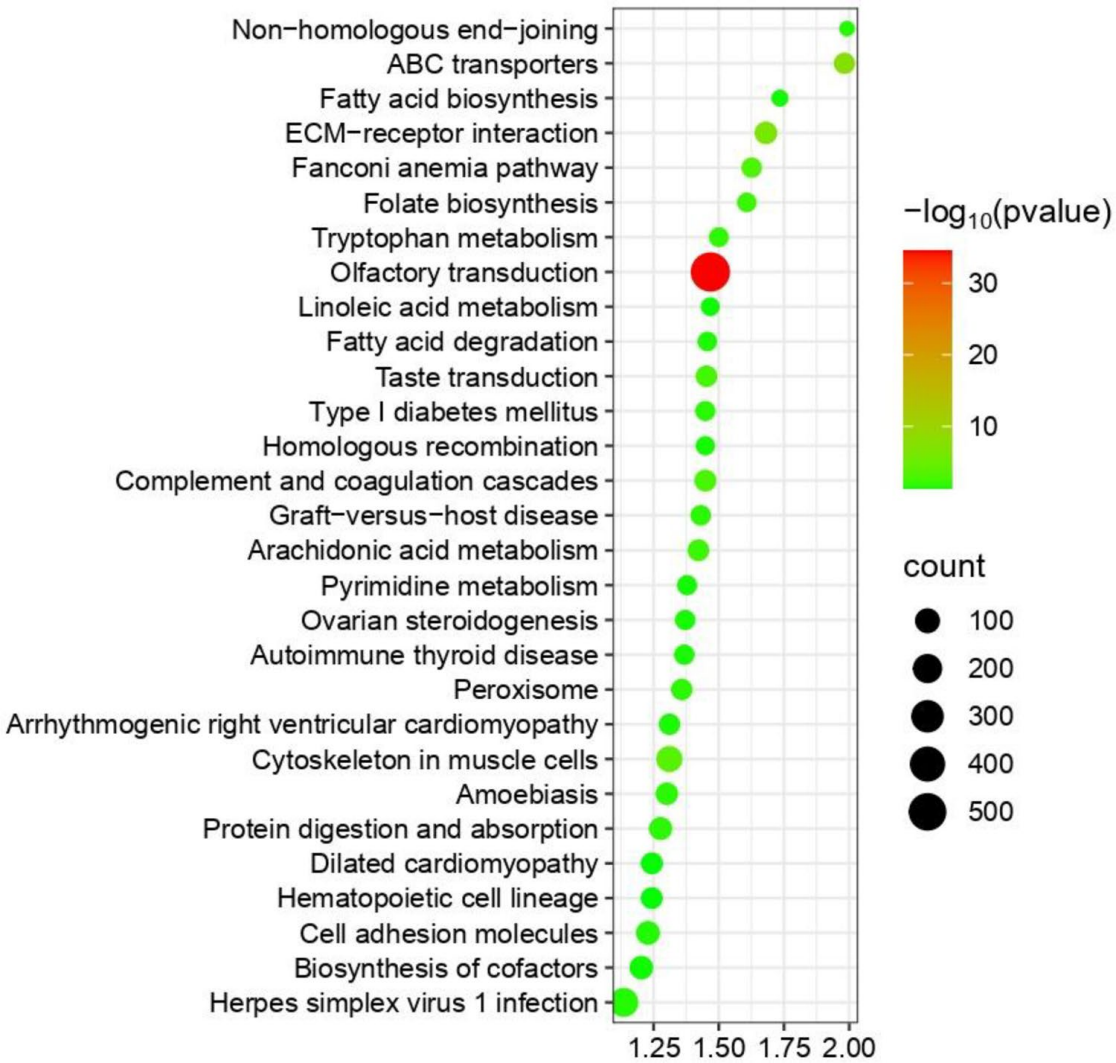
KEGG pathways involvement of genes having high impact variants in Kutta sheep. The size of circles indicates the number of genes. The color indicates P-values. Size of dots indicate the number of genes involved in each of the KEGG pathways. The highest number of genes were involved in Olfactory transduction with the lowest *p* value

The KEGG pathway enrichment analysis shows the functional pathways related to the dataset, organized by their significance (p-value) and the count of enrichment (Fig. 4). The results showed high degree of enrichment in olfactory transduction, tryptophan metabolism, and linoleic acid metabolism, which exhibited the most significant statistical relevance in olfactory transduction. These results suggest that the genes under study are crucially involved in sensory perception, metabolic activities, and signal transduction. In addition, further pathways such as ECM-receptor interaction and fatty acid biosynthesis suggest a role in structural and metabolic regulation. In addition, the analysis focuses on metabolic pathways, such as fatty acid degradation and arachidonic acid metabolism, both of which are critical to energy balance and inflammatory responses. The pathways involved in taste transduction and complement/coagulation cascades provide an example of how sensory and immune processes interact.

### Selection signature of Kutta sheep

**Fig. 5:**
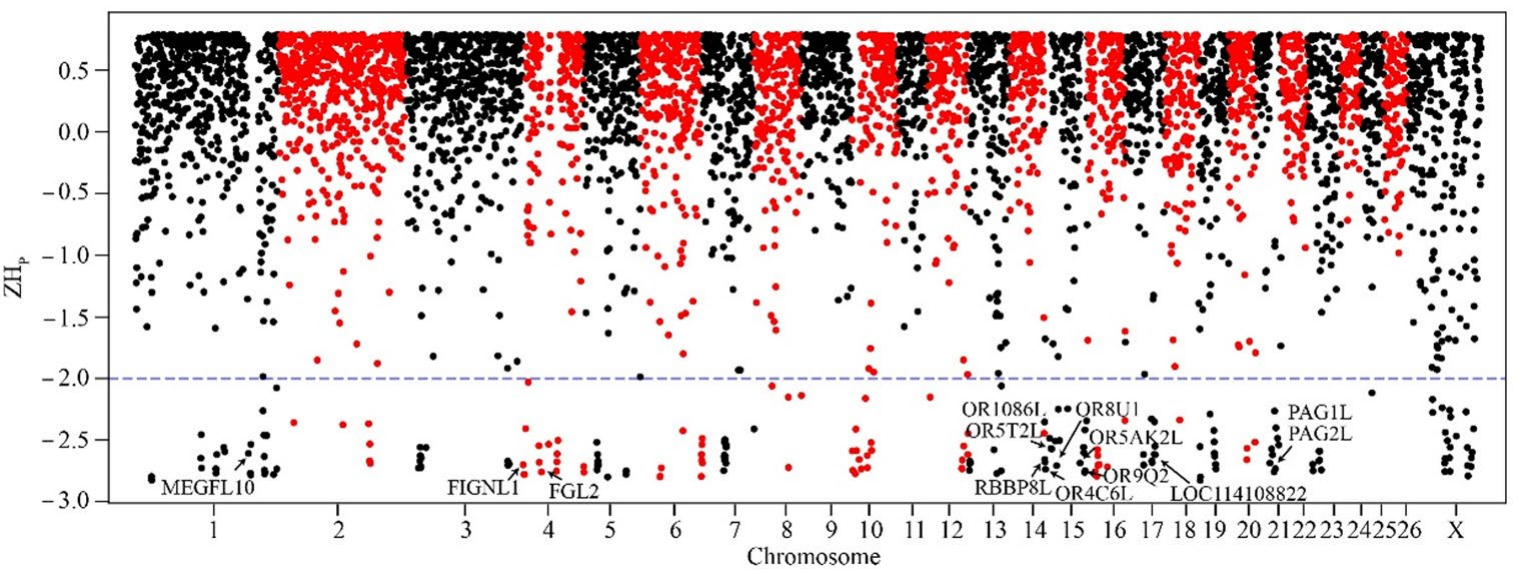
Whole genome selective signature of Kutta sheep evaluated through calculating ZH_P_score. X-axis shows the number of chromosomes and Y-axis shows the ZH_P_ value. Each dot contains 600 kb region of the genome. Genomic regions in the lowest tail of the graph with ZH_P_ value < − 2.0 were selected as potential selective sweep regions, where advantageous alleles have become fixed (homozygosity), reducing diversity. Significant genomic regions showing fixation were further screened for gene loci. Genes showing higher fixation (> 90% homozygosity), presence of non-synonymous variants, their co-localization with previously identified QTLs, and biological functions are labeled. *MEGF10L* multiple epidermal growth factor-like domains protein 10, *FIGNL1* fidgetin like 1, *PAG1L* pregnancy-associated glycoprotein 1-like, *PAG2L* pregnancy-associated glycoprotein 2-like, *OR4C6L* olfactory receptor 4C6-like, *OR1086L* olfactory receptor 1086-like, *OR5AK2L* olfactory receptor 5AK2-like, *OR5T2L* olfactory receptor 5T2-like, *OR9Q2* olfactory receptor 9Q2, *RBBP8L* retinoblastoma binding protein 8-like, *FGL2* Fibrinogen-like protein 2

The whole genome of Kutta sheep was divided into a total number of 5864 windows with an average ZH_P_ score of 1.19E-12 (Fig. 5). The ZH_P_ score ranged from a minimum of − 2.83 to a maximum of 0.79. The genomic windows with ZH_P_ score at the extreme lower end of the plot i.e. below − 2.0 were selected. A total number of 574 peak windows were found below the threshold ZH_P_ score.

These genomic regions contained loci for a total of 1930 genes. The genes were further screened in the VCF file for the SNP density and homozygosity. From these genes, 142 genes were selected with an SNP density > 1%, and with a high percentage (> 90%) of homozygous variants. Further screening for the presence of high impact non-synonymous variants affecting the protein structure narrowed down the list to 96 genes (Supplementary Table 6). These are considered as putative candidate genes under selection and are involved in several biological processes (such as sensory perception of smell and GPCR), molecular functions, cell membrane components, and KEGG pathways (Supplementary Table 7).

After searching for the proximities of these genes with previously known sheep QTLs and their biological relevance, a set of 11 genes was finally identified as selective signature of Kutta sheep (Table 2). Five of these genes were associated with previously known QTLs, while the remaining were selected for the presence of a higher number of non-synonymous variants and their function annotation. Six of these genes were associated with olfactory transduction mechanism with high SNP density.

**Table 2:** Genes identified as selective signature of Kutta sheep, their functions, and fixation values

Gene	Known function	Colocalization with known QTL (ID)	SNP density (%)	Homozygosity (fixation)	Number of high impact variants
MEGF10L	Cell cycle, proliferation, and developmental processes	Milk yield (57709)	1.08	98.8	1
FIGNL1	DNA repair	Fecal egg count (256939)	1.82	100	8
PAG1L	Cell signaling, immune regulation, and T-cell activation		1.70	100	6
PAG2L	Immune regulation, placentogenesis, fetal development		2.05	99.4	18
OR4C6L	Sense of smell locate food sources, permitting survival, identify mates, promoting reproduction, avoid predators	OR1086L and QR5T2L associated with Entropion (3385)	9.58	98.9	10
OR1086L			2.97	100	9
OR5AK2L			4.14	100	2
QR5T2L			13.02	100	4
OR9Q2			3.09	100	3
RBBP8L	DNA damage repair		1.06	100	13
FGL2	Immune regulation, coagulation, and inflammatory responses		1.05	100	6

*MEGF10L* multiple epidermal growth factor-like domains protein 10, *FIGNL1* fidgetin like 1, *PAG1L* pregnancy-associated glycoprotein 1-like, *PAG2L* pregnancy-associated glycoprotein 2-like, *OR4C6L* olfactory receptor 4C6-like, *OR1086L* olfactory receptor 1086-like, *OR5AK2L* olfactory receptor 5AK2-like, *OR5T2L* olfactory receptor 5T2-like, *OR9Q2* olfactory receptor 9Q2, *RBBP8L* retinoblastoma binding protein 8-like, *FGL2* Fibrinogen-like protein 2

**Fig. 6:**
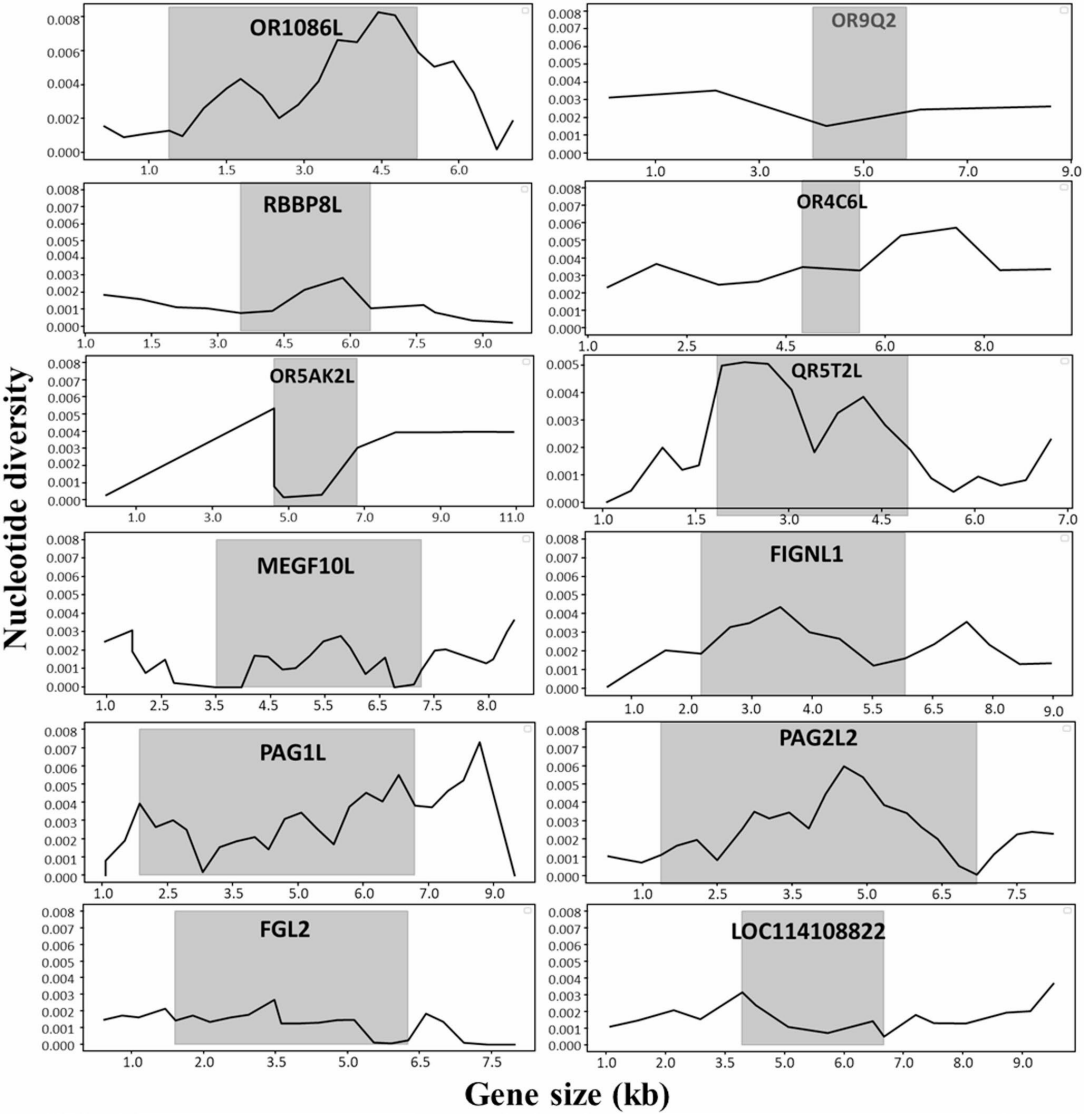
Nucleotide diversity of the genes identified in selective sweep analysis. X-axis represents the gene size in kb and Y-axis represents nucleotide diversity. Low nucleotide diversity values indicate strong selective sweeps where a beneficial allele has reduced diversity. Low nucleotide diversity score + low ZHP score indicate strong selective sweep. The shaded region in each graph shows the gene location, the unshaded regions show upstream and downstream regions. *MEGF10L* multiple epidermal growth factor-like domains protein 10, *FIGNL1* fidgetin like 1, *PAG1L* pregnancy-associated glycoprotein 1-like, *PAG2L* pregnancy-associated glycoprotein 2-like, *OR4C6L* olfactory receptor 4C6-like, *OR1086L* olfactory receptor 1086-like, *OR5AK2L* olfactory receptor 5AK2-like, *OR5T2L* olfactory receptor 5T2-like, *OR9Q2* olfactory receptor 9Q2, *RBBP8L* retinoblastoma binding protein 8-like, *FGL2* Fibrinogen-like protein 2

Overall low nucleotide diversity was observed in all the genes (below 0.008) suggesting fixation in these genes (Fig. 6). Low nucleotide diversity is indicative of high homozygosity in these genes, as these genes were selected for regions of the genome showing extreme fixation/homozygosity. Comparatively lower diversity was observed for *OR9Q2*, *OR4C6L*, *MEGF10L*,* FGL2*, and the uncharacterized protein *LOC114108822*. Small peaks of diversity were observed in the other selective sweep genes.

#### Variation in OR4C6L gene among lowland and highland sheep breeds

**Fig. 7:**
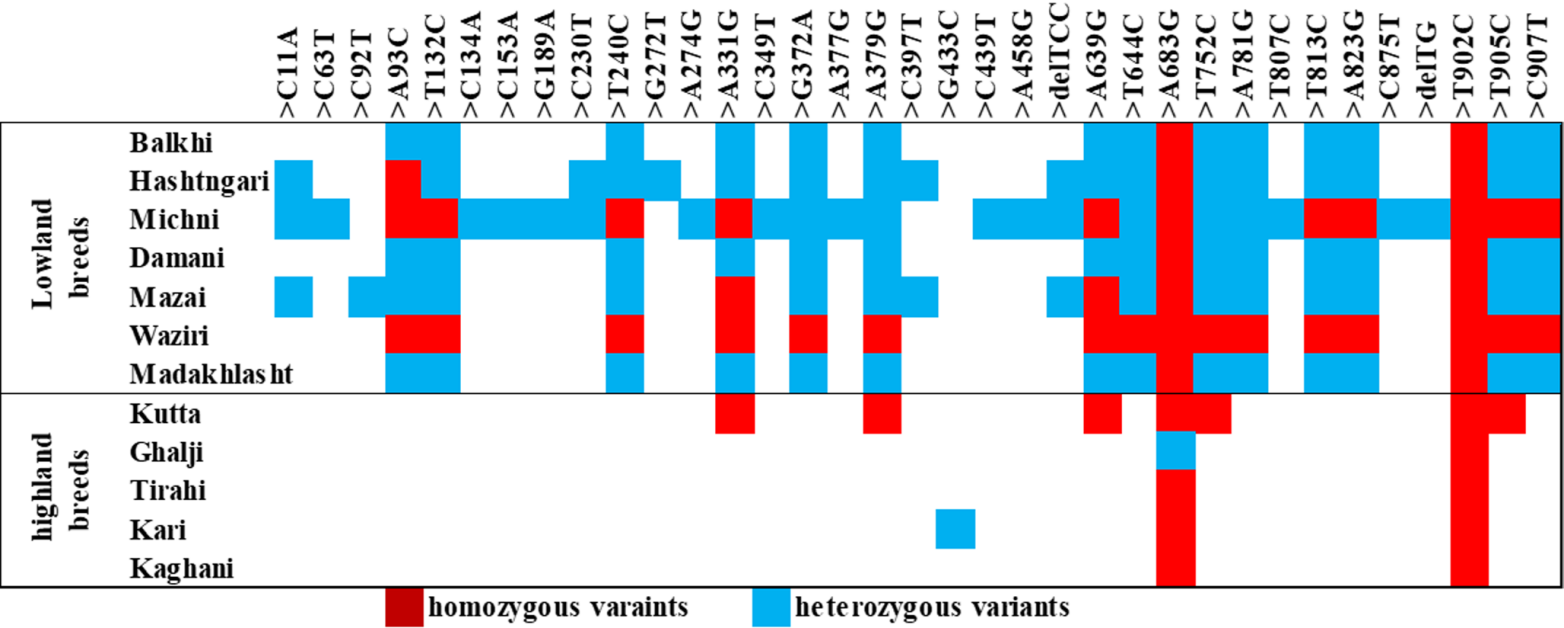
Polymorphism in OR4C6L gene among highland and lowland sheep breeds. Clear allele variation can be seen among lowland and highland sheep breeds at multiple positions in the gene. Nucleotide changes are shown in the top row. Heterozygous variants are shown in blue and homozygous variants are shown in red color

The variant information for the olfactory receptor gene OR4C6L was compared among 12 indigenous sheep breeds (Fig. 7). The results showed significant variations among lowland and highland sheep breeds. The overall structure of 7 lowland sheep breeds was relatively similar to each other; however, it was different from the 5 highland sheep breeds. Relatively more variants were observed in the lowland sheep compared to the highland sheep. Most of the variants in the lowland sheep were heterozygous, while the highland sheep contained homozygous variants, suggesting fixation of this gene in highland sheep.

The diversity estimates for polymorphism in OR4C6L gene in lowland and highland sheep breeds are presented in Table 3. Overall low diversity was observed in highland sheep breeds suggesting fixation. Most of the mutations in highland sheep breeds were significantly deviated from HWE (*p* < 0.05), suggesting possible selection or inbreeding effect. The Tajima D score was below − 2 in lowland sheep breeds suggesting excess of rare alleles and population expansion. In highland sheep breeds the Tajima D score for OR4C6L polymorphism was below − 1.5, which may suggest possible selection signal.

**Table 3:** Diversity parameters for polymorphism in OR4C6L gene in lowland and highland sheep breeds

Diversity parameters	Lowland sheep	highland sheep
HWE	0.60 ± 0.39	0.28 ± 0.48
Shannon index	0.41 ± 0.22	0.09 ± 0.18
Homozygosity	0.60 ± 0.30	0.98 ± 0.04
Mutation frequency	0.35 ± 0.31	0.08 ± 0.22
F_IS_	-0.3 ± 0.26	0.72 ± 0.51
Nucleotide diversity	0.26 ± 0.17	0.05 ± 0.11
Tajima’s D (Simplified)	-5.65 ± 4.60	-1.86 ± 3.57

*HWE* Hardy Weinberg equilibrium, F_IS_ Fisher’s within population inbreeding estimate

## Discussion

Geographical distribution and husbandry practices significantly affect sheep performance and exert a selection pressure on its genome causing adaptation. Different parameters evaluated in the current study may be associated with the performance and genetic architecture of Kutta sheep. Variation in flock size has been found to have a role in sheep’s performance, selection intensity, and grant for household activities (Tadesse Amare et al. 2018). The average flock size for Kutta in the current study was slightly greater than the flock size reported for Ethiopian sheep breeds (20.1 heads per flock) in the sub-alpine area (Gebremichael 2008). Similarly, Abebe et al. (2013) reported smaller flocks of 3.7 ± 2.4 sheep per household in North Western Ethiopia. Tsedeke (2007) also noted an average flock size of 7.4 in the Alaba Zone of Southern region, Ethiopia. However, according to Awgichew (2000) and Samuel (2005) reports, at higher altitudes of 2800–3000 m average flock size is usually above 30 heads, as observed in the current study. Large flock sizes at higher altitudes might be required for better husbandry and protection from predators.

Integrative distribution, production, morphology, and genetic studies provide valuable information regarding breed qualities and future breeding programs (Barillet 2007). Kutta sheep are a distinct breed defined by their morphological traits and ability to adapt to local conditions. Kutta sheep’s morphological traits show significant variation between age and sex groups, which may be due to both developmental and hereditary factors. Indigenous breeds such as Karakul sheep have shown similar patterns in age-related cranial morphology (Jeanjean et al. 2022). These findings imply that age and sex have a major impact on the morphological characteristics of Kutta sheep, most likely as a result of growth-related and hormonal factors suggested in a previous study (Han et al. 2016). Rams of Kutta sheep were more likely to be horned than ewes, suggesting a potential hormonal or genetic basis for this trait. A similar age and sex-related horn development pattern has been noted in breeds such as the Red Maasai sheep (Zonabend König et al. 2016). Together, these results offer insightful information about the morphological and phenotypic variety of Kutta sheep that may help guide conservation and breeding efforts (Ganie 2023).

Genome-wide association analysis provides an opportunity to study the genomic regions and mutations which underpin phenotypic and production traits. The total genomic variants observed in Kutta sheep were similar to the number of variants reported for Asiatic mouflon and improved breeds (Eydivandi et al. 2021). Comparatively, higher number of variants have been reported for Tibetan breeds (Zhang et al. 2021) and Turkish native sheep breeds (Argun Karsli et al. 2024). Several genes have been identified as selective signature of Kutta sheep in the current study, that have roles in different biological and molecular functions such as sensory perceptions, G-protein coupled receptor signaling pathways, and cell membrane components. Some of these genes might be involved in adaptation to the cold environment and better oxygen utilization. In a previous study, differentially expressed genes involved in G-protein coupled receptor signaling pathways have been identified to have role in cold adaptation in Mongolian sheep (Meng et al. 2025). A large number of genes involved in protein binding identified showing mutations in the current study may have a role in oxygen utilization at higher altitudes in Kutta sheep. Previous studies have identified similar genes involved in oxygen utilization under hypoxic conditions in different sheep breeds (An et al. 2023; He et al. 2023; Tian et al. 2025).

Selective signatures provide important insights into genes and pathways associated with evolution and adaptation under a specific environment. Out of the 11 highly important selective signature genes identified in the current study, most are related to olfactory receptors which play an important role in reproduction and adaptation. Different breeds have shown different selective signatures based on the environment where they have evolved and their husbandry practices. In a previous study, we have identified genes for reproductive behavior, milk production, and body size under selection pressure in Mazai and Waziri sheep breeds from Waziristan, Pakistan (Ibrahim et al. 2026). Similarly, Zhao et al. (2020) reported nine genes linked to fat deposition in tails of Chinese sheep breeds. Manzari et al. (2019) found potential genes under selection pressure for skeletal system and tail development, sugar and energy metabolisms, growth, reproduction, immune as well as nervous system characteristics in Iranian sheep breeds.

Edea et al. (2019) identified 5 genes associated with altitude adaptation in Ethiopian sheep breeds. Similarly, Saadatabadi et al. (2021) identified genes associated with wool production; fiber length; development of hair follicle; fiber growth and development; and the ability to withstand dry and hot conditions as selective signature in Iranian sheep breeds. Xu et al. (2021) detected strong selection signatures genes related to fertility, ear size, body size, and tail fat deposition in domestic sheep from different parts of the world. Abied et al. (2020) identified strong selection signatures in genes for body size, ear size, and tail fat deposition. Interestingly, a separate set of genes have been identified as selective signatures of different sheep breeds, which do not coincide with the gene set identified in the current study.

Genes for olfactory transduction have been identified under selection pressure in different sheep breeds in previous studies. Among the different olfactory genes revealed in *OAR15* in Latxa sheep, they confirm evidence of the effect of current genetic selection for resistance against scrapie (Ruiz-Larrañaga et al. 2018). They also identified this selection process, affecting a set of genes related to reproduction such as *ZNF366* and *ESR1*. Similarly, olfactory receptor genes on chromosome 2 have been identified under strong selection in Brazilian sheep breeds (Paim et al. 2022). Those studies report the association of olfactory receptor genes mainly with disease resistance and reproduction. However, the current study suggests that selection of the olfactory receptor genes in Kutta sheep might have a role in highland-specific adaptation by improving the sense of smell for locating food and protecting the animals from predators. Changes in smell perception due to olfactory receptor diversity has been identified as important factor in lamb-dam connection, survival, and adaptation to ecological habitat in sheep and other animals (Dwyer et al. 2016; Silva et al. 2020). Conversely, studies on animals’ adaptation to high altitudes have shown loss of function in olfactory receptor genes possibly contributed to reduced odorant diversity at such altitudes (Zhou et al. 2022; Graham et al. 2025). Therefore, further functional studies are required to identify molecular mechanisms of olfactory transduction in adaptation to high altitude.

This study provides a comprehensive data set integrating performance, morphology and genome wide selective signature in endangered Kutta sheep. The identification of olfactory receptor genes might be a key adaptive signature for mountain survival. The identification of a large number of genes showing fixation needs further exploration to identify their roles associated with the production traits of Kutta sheep. However, the study is limited by certain aspects that need to be considered before using these results in policy making.

Sample size was limited, which may not be representative of the entire population and may have constraints on statistical outcomes. Pooling DNA samples for sequencing may cause unequal distribution of individual DNA samples; furthermore, it limits population structure and allele frequency analyses. It also limits the analyses where multiple samples are required e.g. FST and XP-CLR calculation, so the region under selective sweep identified in zHP score could not be validated or confirmed with other methods. No rams were included for DNA sampling. This may bias variant calling and selection signature detection, given sexual dimorphism in horn development and adaptive traits. Further study is required to validate the key loci in a large population using individual genotyping. Role of olfactory receptor genes in adaptation to high altitude need to be validated though gene expression analysis, protein function studies, and *in vitro* studies.

## Conclusions

The geographical distribution and performance of Kutta sheep in mountainous region is the result of years of selection which is reflected in its genome. A high number of genomic variants were identified in Kutta sheep, 0.22% of which were exon variants. Highly mutated genes are mostly associated with g-protein coupled receptors, olfactory transduction, and membrane components. The Selective sweep analysis identified a set of 11 genes which might be associated with breeding and reproductive performance of Kutta sheep in their habitat. Selection of olfactory transduction genes may have enabled Kutta sheep to survive and thrive in mountainous regions by improving their sense of smell. Further validation of these results is required at expression and protein level to identify the role of olfactory receptors in sensory perception of smell and their role in improving breeding and survivability in mountainous sheep.

## Supplementary Information

Below is the link to the electronic supplementary material.

Supplementary Material 1

Supplementary Material 2

## Data Availability

The data that support the findings of this study are available from the corresponding author upon reasonable request.
