# Supplementary material for: Genome sequencing reveals genes under selection for olfactory transduction in highland sheep
Source: Mol Genet Genomics. 2026 Jul 14;301(1):155. doi: 10.1007/s00438-026-02477-1 (PMC13369732; doi:10.1007/s00438-026-02477-1)
Supplement: Supplementary file 1 — Supplementary Material 1 [file 438_2026_2477_MOESM1_ESM.docx]

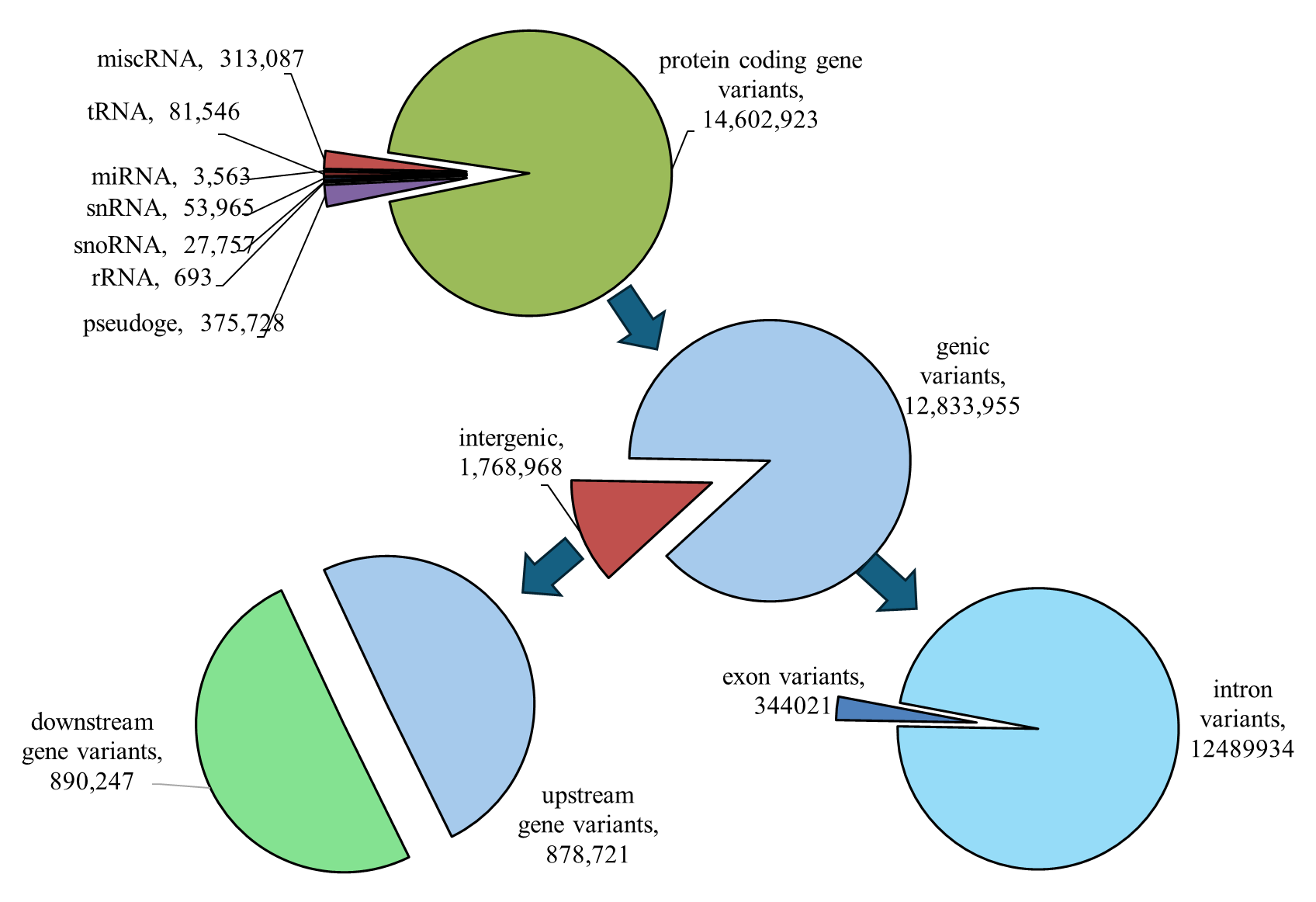


Supplementary Figure 1: Total genomic variants in Kutta sheep’s genome compared to the reference genome (Oar_rambouillet_v1.0, RefSeq assembly accession No. GCF_002742125.1). The highest number of variants were identified in the protein coding genes.
