# Supplementary material for: Genome sequencing reveals genes under selection for olfactory transduction in highland sheep
Source: Mol Genet Genomics. 2026 Jul 14;301(1):155. doi: 10.1007/s00438-026-02477-1 (PMC13369732; doi:10.1007/s00438-026-02477-1)
Supplement: Supplementary file 2 — Supplementary Material 2 [file 438_2026_2477_MOESM2_ESM.docx]

**Supplementary table 1: Management practices adopted for Kutta sheep flocks in Kalam area and productive and reproductive performance flocks under these practices.**

| **Flock management and performance** | **Values** |
| --- | --- |
| Farming system | Transhumant (82.6%), Sedentary (17.4%) |
| Number of adult ewes | 16.7 ± 1.9 |
| Number of Rams | 1.6 ± 0.2 |
| Number of post-weaning stock | 6.5 ± 0.9 |
| Number of pre-weaning stock | 4.5 ± 0.7 |
| Flock size (number) | 29.4 ± 3.5 |
| Ewes to ram ratio | 10.5 ± 1.0 |
| Breeding practice | Continuous (91.3%), Restricted (8.7%) |
| Age of ram (months) | 26.8 ± 1.3 |
| Lambing month | March (39.1%), April (52.2%), May (8.7%) |
| Number of ewes lambed | 13.5 ± 1.7 |
| Lambing efficiency (%) | 77.2 ± 2.6 |
| Percent ewes lambed twice/annum | 31.6 ± 2.2 |
| Age at first lambing (months) | 18.6 ± 0.3 |
| Gestation period (days) | 157.4 ± 0.8 |
| Weaning process | Self (91.3%), Forced (8.7%) |
| Weaning age (months) | 3.3 ± 0.1 |
| Grazing hrs | 7.6 ± 0.2 |
| Feed supplementation | Wheat barn, soyabean cake |
| Vaccination | FMD, sheep pox, Enterotoxemia |
| Deworming | Albendazole, ivermectin, levamisole |
| Ewe price (PKR) | 20000 ± 288 |
| Ram price (PKR) | 25000 ± 466 |
| Lamb price (PKR) | 30000 ± 795 |

Key: FMD = foot and mouth disease; PKR = Pakistani rupee

**Supplementary Table 2: differences in the body morphometry of Rams, ewes, and lambs of Kutta sheep.**

| **Parameters** | **Sheep category** | | | **F-value** | **P-value** |
| --- | --- | --- | --- | --- | --- |
|  | **Ewe** | **Ram** | **Lamb** |  |  |
| Head Length | 20.0 ± 1.2 | 21.8 ± 2.2 | 12.5 ± 2.5 | 2.28 | 0.124 |
| Mouth Width | 24.0 ± 0.9^b^ | 24.2 ± 1.8^b^ | 15.5 ± 1.5^a^ | 3.53 | 0.045 |
| Neck Length | 22.4 ± 1.2^b^ | 22.2 ± 1.4^b^ | 11.0 ± 0.0^a^ | 5.53 | 0.011 |
| Neck Girth | 32.0 ± 2.0^b^ | 34.3 ± 1.9^b^ | 19.5 ± 2.5^a^ | 3.63 | 0.043 |
| Body Length | 58.9 ± 2.6^b^ | 58.4 ± 3.2^b^ | 27.0 ± 2.0^a^ | 9.52 | 0.001 |
| Heart girth | 75.6±3.7^b^ | 77.7±4.4^b^ | 32.5±5.5^a^ | 9.27 | 0.001 |
| Belly girth | 85.4 ± 3.7^b^ | 86.1 ± 4.4^b^ | 35.0 ± 2.0^a^ | 12.53 | 0.000 |
| Heart depth | 26.2 ± 1.0 | 26.5 ± 1.2 | 21.0 ± 0.0 | 1.73 | 0.202 |
| Belly depth | 31.0 ± 1.2 | 30.5 ± 1.3 | 24.0 ± 0.0 | 2.28 | 0.124 |
| body height at withers | 57.8 ± 2.6 | 56.0 ± 2.9 | 34.0 ± 2.0 | 5.42 | 0.071 |
| body height at rump | 62.3 ± 2.8^b^ | 62.2 ± 3.4^b^ | 37.5 ± 1.5^a^ | 5.02 | 0.036 |
| Rump Width at front | 17.9 ± 1.1^b^ | 18.0 ± 1.1^b^ | 10.5 ± 1.5^a^ | 2.96 | 0.004 |
| Rump Width at back | 24.6 ± 1.4 | 24.3 ± 1.5 | 14.0 ± 2.0 | 3.82 | 0.901 |
| Rump Length | 61.4 ± 2.4 | 61.1 ± 3.6 | 33.5 ± 5.5 | 7.26 | 0.107 |
| Tail length | 18.0 ± 0.8 | 17.3 ± 1.3 | 17.5 ± 1.5 | 0.10 | 0.167 |
| Tail diameter at base | 13.4 ± 0.9 | 11.2 ± 0.9 | 8.5 ± 0.5 | 2.47 | 0.275 |
| Tail diameter at midpoint | 9.4 ± 0.8^a^ | 7.77 ± 0.7^a^ | 6.0 ± 0.0^a^ | 1.94 | 0.012 |
| Tail diameter at end | 5.9 ± 0.6^a^ | 4.8 ± 0.6^a^ | 3.5 ± 0.5^a^ | 1.37 | 0.016 |

Key: Superscripts on the value show pairwise differences among sheep categories (PostHoc Tukey’s HSD test, mean differences is significant at the 0.05 level). degree of freedom = 25

**Supplementary Table 3: Differences in the body morphometry of Kutta sheep of different age groups.**

| **Parameters** | **Sheep category** | | | **F-value** | **P-value** |
| --- | --- | --- | --- | --- | --- |
|  | **1 and less** | **1-3 years** | **>3 years** |  |  |
| Head Length | 13.8 ± 1.0^a^ | 19.2 ± 1.2^b^ | 27.5 ± 0.4^c^ | 18.55 | 0.000 |
| Mouth Width | 17.6 ± 1.2^a^ | 24.6 ± 1.2^b^ | 25.3 ± 0.8^b^ | 6.67 | 0.000 |
| Neck Length | 15.8 ± 1.9^a^ | 21.6 ± 1.2^ab^ | 25.6 ± 1.2^b^ | 6.62 | 0.005 |
| Neck Girth | 25.6 ± 2.6^a^ | 30.0 ± 1.2^a^ | 41.5 ± 2.8^b^ | 13.14 | 0.005 |
| Body Length | 39.8 ± 5.3^a^ | 57.0 ± 2.3^b^ | 68.1 ± 2.0^c^ | 13.43 | 0.000 |
| Heart girth | 52.0 ± 8.3^a^ | 71.2 ± 2.0^b^ | 95.0 ± 4.5^c^ | 20.57 | 0.000 |
| Belly girth | 61.0 ± 10.0^a^ | 80.8 ± 2.7^b^ | 101.0 ± 4.2^c^ | 11.46 | 0.000 |
| Heart depth | 23.4 ± 1.0^a^ | 26.8 ± 1.2^b^ | 26.0 ± 0.4^c^ | 1.34 | 0.000 |
| Belly depth | 26.4 ± 1.0 | 30.9 ± 1.3 | 32.0 ± 1.2 | 2.67 | 0.281 |
| body height at withers | 43.4 ± 4.2 | 54.9 ± 2.1 | 66.5 ± 3.2 | 9.95 | 0.091 |
| body height at rump | 46.6 ± 3.9^a^ | 60.0 ± 2.4^b^ | 72.6 ± 3.1^c^ | 11.36 | 0.000 |
| Rump Width at front | 12.8 ± 1.1^a^ | 16.9 ± 0.8^a^ | 22.3 ± 1.3^b^ | 11.87 | 0.001 |
| Rump Width at back | 17.2 ± 1.5^a^ | 23.9 ± 1.3^b^ | 28.6 ± 0.7^b^ | 8.90 | 0.000 |
| Rump Length | 44.8 ± 5.1 | 59.2 ± 2.3 | 71.0 ± 1.6 | 11.81 | 0.145 |
| Tail length | 16.2 ± 0.7 | 17.3 ± 1.0 | 20.0 ± 0.4 | 2.10 | 0.446 |
| Tail diameter at base | 10.4 ± 0.8 | 12.8 ± 1.0 | 12.5 ± 1.6 | 0.83 | 0.306 |
| Tail diameter at midpoint | 6.8 ± 0.3 | 9.06 ± 0.7 | 8.83 ± 1.3 | 1.25 | 0.442 |
| Tail diameter at end | 4.2 ± 0.3^a^ | 5.6 ± 0.6^a^ | 5.8 ± 1.1^a^ | 0.85 | 0.001 |

Key: Superscripts on the value show pairwise differences among sheep categories (PostHoc Tukey’s HSD test, mean differences is significant at the 0.05 level). degree of freedom = 25

**Supplementary Table 4: Color percentages of body different parts of Kutta sheep**

|  | **Black** | **black and white** | **brown** | **brown and white** | **white** |
| --- | --- | --- | --- | --- | --- |
| Ear color | 34.6 | 11.5 | 15.4 | 19.2 | 19.2 |
| Neck color | 42.3 | - | 19.2 | 3.8 | 34.6 |
| Face color | - | - | 7.7 | - | - |
| Loin color | 42.3 | - | 23.1 | - | 34.6 |
| Back color | 38.5 | - | 15.4 | 11.5 | 34.6 |
| Limb color | 34.6 | - | 19.2 | - | 46.2 |
| Tail color | 30.8 | - | 19.2 | - | 50 |
| Forehead color | - | - | - | - | 11.5 |
| Belly color | 38.5 | 3.8 | 11.5 | - | 30.8 |

**Supplementary Table 5: Total variants in genes encoding different RNAs and pseudogenes**

| **Variant type** | **Mi**  **RNA** | **Misc RNA** | **rRNA** | **Sno RNA** | **Sn RNA** | **tRNA** | **Pseudogenes** |
| --- | --- | --- | --- | --- | --- | --- | --- |
| Coding transcript variants |  |  |  |  |  | 573 | 618 |
| Upstream gene variant | 1,829 | 28,346 | 329 | 13,755 | 26,198 | 39,540 | 116,204 |
| Non-coding transcript exon variant |  |  |  |  |  |  | 24,621 |
| Non-coding transcript variant |  | 258,446 | 5 | 248 | 733 | 114 | 5,704 |
| Intron variant |  |  |  |  |  | 9 | 109,479 |
| Downstream gene variant | 1,734 | 26,295 | 359 | 13,754 | 27,034 | 41,310 | 119,102 |

Key: Mi RNA = micro RNA, Misc RNA = miscellaneous RNA, rRNA = ribosomal RNA, Sno RNA = small nucleolar RNA, Sn RNA = small nuclear RNA, tRNA = transfer RNA.

**Supplementary Table 6: the identified genes under selective sweep, their coordinates and fixation percentages**

| **Chrom** | **Gene** | **gene symbol** | **start POS** | **end POS** | **SNPs (count)** | **Homozygosity/fixation%** | **Protein length** | **non-syn variants** | **QTL association** |
| --- | --- | --- | --- | --- | --- | --- | --- | --- | --- |
| 1 | ATP synthase-coupling factor 6 | ATP5PF | 139294045 | 139302458 | 98 | 0.98 | 108 | 1 |  |
| 1 | 60S ribosomal protein L22-like 1 | RPL22L1 | 235719688 | 235724074 | 84 | 1.00 | 122 | 1 |  |
| 1 | multiple epidermal growth factor-like domains protein 10 | LOC106990882 | 237635904 | 237644169 | 89 | 0.99 | 118 | 1 | Milk yield |
| 1 | LOW QUALITY PROTEIN: ubiquitin carboxyl-terminal hydrolase BAP1-like | LOC101116449 | 242676241 | 242678448 | 28 | 1.00 | 728 | 8 |  |
| 1 | ADP-ribosylation factor-like protein 14 | ARL14 | 247478837 | 247479857 | 33 | 1.00 | 192 | 1 |  |
| 1 | methyltransferase-like protein 6 isoform X1 | METTL6 | 296039460 | 296055048 | 177 | 1.00 | 284 | 1 |  |
| 3 | gastrokine-3-like | LOC101108901 | 41217486 | 41223606 | 74 | 1.00 | 181 | 1 |  |
| 4 | protein cordon-bleu-like | LOC106990241 | 5976042 | 6011088 | 397 | 1.00 | 912 | 8 |  |
| 4 | fidgetin-like protein 1 | FIGNL1 | 6627435 | 6631655 | 77 | 1.00 | 677 | 8 | fecal egg count after nematode challenge |
| 4 | fibroleukin | FGL2 | 49563133 | 49566955 | 40 | 1.00 | 441 | 6 |  |
| 4 | leucine-rich single-pass membrane protein 1 | LSMEM1 | 62702069 | 62712644 | 124 | 0.99 | 128 | 1 |  |
| 4 | neuropeptide VF precursor | NPVF | 77511001 | 77514323 | 39 | 1.00 | 196 | 1 |  |
| 6 | neuropeptide-like protein C4orf48 homolog | C6H4orf48 | 128618792 | 128620512 | 32 | 1.00 | 100 | 1 |  |
| 7 | C2 calcium-dependent domain-containing protein 4B | C2CD4B | 49131589 | 49133293 | 44 | 1.00 | 468 | 3 |  |
| 7 | E3 ubiquitin-protein ligase TRIM69 | TRIM69 | 107587310 | 107604893 | 178 | 0.99 | 536 | 4 |  |
| 12 | serine/threonine-protein kinase PLK1-like | LOC114117278 | 70343869 | 70344869 | 31 | 1.00 | 262 | 1 |  |
| 12 | protein RD3 | RD3 | 75420498 | 75423285 | 59 | 1.00 | 195 | 1 |  |
| 14 | uncharacterized protein LOC114117956 | LOC114117956 | 71067807 | 71069290 | 20 | 1.00 | 352 | 1 |  |
| 15 | sperm-egg fusion protein Juno | IZUMO1R | 1033760 | 1035925 | 47 | 1.00 | 243 | 2 |  |
| 15 | neutrophil collagenase | MMP8 | 6145143 | 6159411 | 152 | 0.97 | 470 | 1 |  |
| 15 | DNA endonuclease RBBP8-like | LOC101121879 | 12217259 | 12220274 | 32 | 1.00 | 899 | 13 |  |
| 15 | nicotinamide N-methyltransferase | NNMT | 27355996 | 27361792 | 85 | 1.00 | 229 | 2 |  |
| 15 | uncharacterized protein C11orf71 homolog | C15H11orf71 | 27471684 | 27472325 | 15 | 1.00 | 132 | 1 |  |
| 15 | probable inactive 1-aminocyclopropane-1-carboxylate synthase-like protein 2 | ACCSL | 80316432 | 80336336 | 280 | 0.96 | 611 | 4 |  |
| 15 | 1-aminocyclopropane-1-carboxylate synthase-like protein 1 isoform X1 | ACCS | 80338574 | 80359062 | 234 | 1.00 | 509 | 6 |  |
| 15 | muscarinic acetylcholine receptor M4 | CHRM4 | 82661269 | 82664236 | 48 | 1.00 | 541 | 2 |  |
| 15 | olfactory receptor 4B1-like | LOC101122813 | 84197404 | 84198336 | 40 | 1.00 | 310 | 3 |  |
| 15 | olfactory receptor 4C12-like | LOC101122303 | 84380560 | 84381490 | 84 | 1.00 | 309 | 2 |  |
| 15 | olfactory receptor 4C46-like isoform X1 | LOC101122549 | 84384692 | 84387529 | 80 | 1.00 | 309 | 3 |  |
| 15 | olfactory receptor 5AL1-like | LOC101113791 | 84505706 | 84506647 | 24 | 1.00 | 313 | 3 |  |
| 15 | olfactory receptor 5D13-like | LOC101120558 | 84607321 | 84608289 | 12 | 1.00 | 322 | 3 |  |
| 15 | olfactory receptor 4A47-like | LOC105613588 | 84771174 | 84772091 | 36 | 1.00 | 305 | 4 |  |
| 15 | olfactory receptor 140-like | LOC101104270 | 84804472 | 84805398 | 62 | 1.00 | 308 | 2 |  |
| 15 | olfactory receptor 140-like | LOC101104526 | 84830420 | 84831346 | 66 | 1.00 | 308 | 4 |  |
| 15 | olfactory receptor 4C6-like | LOC101104781 | 84857155 | 84858084 | 89 | 0.99 | 309 | 10 |  |
| 15 | olfactory receptor 4C11-like | LOC101105276 | 84887480 | 84888391 | 14 | 1.00 | 303 | 2 |  |
| 15 | olfactory receptor 1052-like | LOC114118325 | 85227229 | 85228170 | 33 | 1.00 | 312 | 3 |  |
| 15 | olfactory receptor 1052-like | LOC105602384 | 85236278 | 85237216 | 44 | 1.00 | 312 | 1 |  |
| 15 | olfactory receptor 8I2 | LOC101102262 | 85249263 | 85250192 | 31 | 1.00 | 309 | 8 |  |
| 15 | olfactory receptor 8H1-like | LOC101106793 | 85268331 | 85269278 | 37 | 1.00 | 315 | 3 |  |
| 15 | olfactory receptor 5T2-like | LOC114118545 | 85308883 | 85309958 | 86 | 1.00 | 318 | 6 |  |
| 15 | olfactory receptor 1086-like | LOC101107550 | 85326836 | 85333531 | 199 | 1.00 | 387 | 9 | Entropion |
| 15 | olfactory receptor 5T2-like | LOC101108078 | 85335756 | 85336831 | 140 | 1.00 | 318 | 4 | Entropion |
| 15 | olfactory receptor 8J2-like | LOC101102512 | 85353327 | 85354274 | 31 | 1.00 | 315 | 0 | Entropion |
| 15 | olfactory receptor 8K3-like | LOC101109389 | 85393961 | 85394902 | 44 | 1.00 | 313 | 5 |  |
| 15 | olfactory receptor 8K3-like | LOC101109644 | 85405632 | 85406573 | 32 | 0.97 | 313 | 4 |  |
| 15 | olfactory receptor 8K3-like | LOC101109909 | 85416555 | 85417496 | 55 | 1.00 | 313 | 5 |  |
| 15 | olfactory receptor 8K3-like | LOC101110175 | 85425859 | 85426800 | 24 | 1.00 | 313 | 3 |  |
| 15 | olfactory receptor 8K3-like | LOC101110447 | 85440111 | 85441052 | 35 | 0.91 | 313 | 5 |  |
| 15 | olfactory receptor 1052 | LOC101111227 | 85461613 | 85462551 | 11 | 1.00 | 312 | 1 |  |
| 15 | olfactory receptor 8J3-like | LOC101112513 | 85652110 | 85653057 | 18 | 1.00 | 315 | 2 |  |
| 15 | olfactory receptor 8J3-like | LOC101112763 | 85662974 | 85667507 | 83 | 1.00 | 350 | 4 |  |
| 15 | olfactory receptor 8U1 | LOC101113273 | 85690036 | 85695864 | 89 | 0.99 | 324 | 3 | Entropion |
| 15 | olfactory receptor 1038-like | LOC101114305 | 85768076 | 85769032 | 19 | 0.95 | 318 | 2 |  |
| 15 | olfactory receptor 5AP2 | LOC101104008 | 85948753 | 85949706 | 60 | 1.00 | 317 | 6 |  |
| 15 | olfactory receptor 1019 | LOC101104268 | 85957454 | 85958387 | 38 | 1.00 | 310 | 2 |  |
| 15 | olfactory receptor 2G3-like | LOC101115825 | 85971649 | 85972584 | 11 | 1.00 | 311 | 1 |  |
| 15 | olfactory receptor 8H1-like | LOC101116861 | 86197793 | 86198740 | 24 | 1.00 | 315 | 2 |  |
| 15 | olfactory receptor 5AK2-like | LOC101118383 | 86500902 | 86503491 | 41 | 1.00 | 343 | 2 |  |
| 15 | putative olfactory receptor 5AK3 | LOC101118639 | 86518199 | 86519128 | 23 | 1.00 | 309 | 1 |  |
| 15 | olfactory receptor 5AK2-like | LOC101119160 | 86547994 | 86548935 | 39 | 1.00 | 313 | 3 |  |
| 15 | olfactory receptor 6Q1 | LOC101106879 | 87497227 | 87499436 | 44 | 1.00 | 317 | 2 |  |
| 15 | olfactory receptor 9I1 | LOC101107133 | 87557348 | 87558586 | 52 | 1.00 | 314 | 1 |  |
| 15 | olfactory receptor 9I1-like | LOC101102929 | 87581449 | 87582390 | 25 | 1.00 | 313 | 5 |  |
| 15 | olfactory receptor 9Q2 | LOC101103181 | 87646268 | 87647174 | 28 | 1.00 | 301 | 3 |  |
| 15 | olfactory receptor 5B3-like | LOC101105687 | 87941980 | 87942927 | 38 | 1.00 | 314 | 7 |  |
| 15 | olfactory receptor 5B3-like | LOC101105947 | 87954047 | 87955000 | 40 | 0.98 | 316 | 5 |  |
| 15 | olfactory receptor 5B2-like | LOC101106460 | 87974858 | 87975802 | 73 | 1.00 | 314 | 8 |  |
| 15 | olfactory receptor 5B2-like | LOC101106967 | 87988238 | 87990440 | 52 | 1.00 | 323 | 1 |  |
| 15 | olfactory receptor 5B2-like | LOC101107385 | 88004699 | 88005637 | 38 | 0.97 | 312 | 3 |  |
| 15 | olfactory receptor 5B12-like | LOC101108776 | 88230537 | 88231484 | 51 | 1.00 | 314 | 7 |  |
| 15 | olfactory receptor 5B12-like | LOC101109300 | 88252053 | 88253006 | 19 | 1.00 | 317 | 2 |  |
| 15 | ciliary neurotrophic factor | CNTF | 88383854 | 88386394 | 34 | 1.00 | 199 | 2 |  |
| 16 | granzyme A | LOC101117107 | 25396012 | 25408013 | 163 | 1.00 | 260 | 1 |  |
| 17 | uncharacterized protein LOC114108822 | LOC114108822 | 66674004 | 66677810 | 59 | 1.00 | 325 | 11 |  |
| 21 | FAD-dependent oxidoreductase domain-containing protein 1 | FOXRED1 | 30620995 | 30628899 | 96 | 1.00 | 486 | 1 |  |
| 21 | pepsin A-like | LOC114110083 | 38542954 | 38545279 | 62 | 1.00 | 120 | 6 |  |
| 21 | pregnancy-associated glycoprotein 2-like | LOC101122394 | 38599892 | 38608637 | 91 | 1.00 | 380 | 2 |  |
| 21 | pregnancy-associated glycoprotein 2-like | LOC101122824 | 38620872 | 38629845 | 130 | 1.00 | 376 | 11 |  |
| 21 | pregnancy-associated glycoprotein 1-like | LOC101123161 | 38634965 | 38644764 | 167 | 1.00 | 359 | 6 |  |
| 21 | pregnancy-associated glycoprotein 1-like | LOC101122899 | 38661912 | 38670658 | 111 | 1.00 | 377 | 5 |  |
| 21 | pregnancy-associated glycoprotein 1-like | LOC101102275 | 39492297 | 39501083 | 122 | 1.00 | 396 | 10 |  |
| 21 | protein phosphatase 1A-like | LOC101103023 | 39594996 | 39602842 | 87 | 1.00 | 322 | 4 |  |
| 21 | pregnancy-associated glycoprotein 2-like | LOC101108872 | 41584336 | 41592228 | 162 | 0.99 | 314 | 18 |  |
| 21 | pregnancy-associated glycoprotein 2-like | LOC101105611 | 41617568 | 41626970 | 102 | 1.00 | 386 | 1 |  |
| 21 | 60S ribosomal protein L10-like | LOC101110719 | 42444266 | 42446875 | 27 | 1.00 | 117 | 6 |  |
| 21 | secretoglobin family 1D member-like | LOC101110099 | 42560526 | 42563304 | 30 | 1.00 | 101 | 1 |  |
| 21 | SAC3 domain-containing protein 1 | SAC3D1 | 45057318 | 45060521 | 50 | 1.00 | 358 | 2 |  |
| 21 | ribonuclease H2 subunit C | RNASEH2C | 45677525 | 45678755 | 36 | 1.00 | 165 | 2 |  |
| 21 | AP-5 complex subunit beta-1 | AP5B1 | 45688386 | 45691710 | 43 | 1.00 | 877 | 5 |  |
| 21 | protein preY | LOC106990260 | 46266563 | 46267005 | 14 | 0.93 | 116 | 0 |  |
| 21 | NADH dehydrogenase [ubiquinone] flavoprotein 1 | NDUFV1 | 47214331 | 47220095 | 71 | 1.00 | 464 | 1 |  |
| 21 | double C2-like domain-containing protein gamma | LOC101121557 | 47220827 | 47224715 | 78 | 1.00 | 379 | 5 |  |
| 21 | mas-related G-protein coupled receptor member D | MRGPRD | 48077432 | 48078391 | 10 | 1.00 | 319 | 1 |  |
| 21 | mas-related G-protein coupled receptor member F | MRGPRF | 48094043 | 48103342 | 93 | 1.00 | 343 | 1 |  |
| 23 | uncharacterized protein C8orf59 homolog | LOC101115843 | 25315674 | 25316841 | 47 | 1.00 | 127 | 1 |  |

Key: Chrom = chromosome number, POS = position

Supplementary Table 7: involvement of selective sweep genes in biological processes, cellular component and KEGG pathways.

| Category | Term | Pathway | Gene count | PValue | Genes | Fold Enrichment | FDR |
| --- | --- | --- | --- | --- | --- | --- | --- |
| BP_DIRECT | GO:0007608 | sensory perception of smell | 31 | 7.34E-44 | LOC101118639, LOC101110175, LOC101112513, LOC101109389, LOC101109644, LOC101108776, LOC101111227, LOC101106793, LOC101103181, LOC101108078, LOC101107385, LOC101102512, LOC101109909, LOC101119160, LOC101105687, LOC101105947, LOC101114305, LOC101102262, LOC105602384, LOC101110447, LOC101107133, LOC101107550, LOC101106460, LOC101106967, LOC101104008, LOC101104268, LOC101102929, LOC101116861, LOC114118545, LOC114118325, LOC101113273 | 50.53425 | 4.62E-42 |
| BP_DIRECT | GO:0007186 | G protein-coupled receptor signaling pathway | 42 | 4.34E-33 | LOC101118639, LOC101110175, LOC101112513, LOC101109389, LOC101109644, LOC105613588, LOC101108776, MRGPRF, LOC101111227, LOC101106793, LOC101115825, LOC101103181, LOC101108078, LOC101107385, LOC101104270, LOC101102512, LOC101109909, LOC101106879, LOC101119160, LOC101122303, LOC101105687, LOC101122549, LOC101105947, LOC101114305, LOC101102262, LOC101104781, LOC105602384, LOC101110447, LOC101105276, LOC101107133, LOC101107550, LOC101106460, LOC101104526, LOC101106967, LOC101104008, LOC101104268, LOC101102929, LOC101122813, LOC101116861, LOC114118545, LOC114118325, LOC101113273 | 10.50586 | 1.37E-31 |
| BP_DIRECT | GO:0006508 | proteolysis | 8 | 0.010047 | LOC101105611, LOC101108872, LOC101117107, LOC114110083, LOC101102275, LOC101122824, MMP8, LOC101122394 | 3.288072 | 0.210982 |
| BP_DIRECT | GO:0006520 | amino acid metabolic process | 2 | 0.023149 | ACCSL, ACCS | 84.35959 | 0.364592 |
| BP_DIRECT | GO:0009058 | biosynthetic process | 2 | 0.059643 | ACCSL, ACCS | 32.13699 | 0.751505 |
| CC_DIRECT | GO:0016020 | membrane | 45 | 1.11E-15 | LOC101118639, LOC101110175, LOC101112513, LOC101109389, LOC101109644, LOC105613588, LOC101108776, MRGPRF, LOC101111227, LOC101106793, LOC101115825, LOC101103181, LOC101108078, LOC101107385, LOC101104270, LOC101102512, LOC101109909, LOC101106879, LOC101119160, LOC101122303, LOC101105687, LOC101121557, LOC101122549, LOC101105947, LOC101114305, LOC101102262, LOC101104781, LOC105602384, LOC101110447, LOC101105276, LOC101107133, LOC101107550, LOC101106460, LOC101122394, LOC101104526, LOC101106967, LOC101104008, LOC101104268, LOC101102929, LOC101122813, LOC101116861, LOC114118545, LOC101120558, LOC114118325, LOC101113273 | 3.388763 | 4.00E-14 |
| CC_DIRECT | GO:0005886 | plasma membrane | 36 | 3.13E-08 | LOC101118639, LOC101110175, LOC101112513, LOC101109644, CHRM4, LOC105613588, LOC101108776, MRGPRF, LOC101111227, LOC101106793, LOC101103181, LOC101108078, LOC101107385, LOC101104270, LOC101102512, LOC101109909, LOC101119160, LOC101122303, LOC101105687, LOC101122549, LOC101105947, LOC101102262, LOC101104781, LOC105602384, LOC101110447, LOC101105276, LOC101107550, LOC101106460, LOC101104526, LOC101104268, LOC101102929, LOC101122813, LOC101116861, LOC114118545, LOC114118325, LOC101113273 | 2.524408 | 5.63E-07 |
| MF_DIRECT | GO:0005549 | odorant binding | 31 | 5.37E-44 | LOC101118639, LOC101110175, LOC101112513, LOC101109389, LOC101109644, LOC101108776, LOC101111227, LOC101106793, LOC101103181, LOC101108078, LOC101107385, LOC101102512, LOC101109909, LOC101119160, LOC101105687, LOC101105947, LOC101114305, LOC101102262, LOC105602384, LOC101110447, LOC101107133, LOC101107550, LOC101106460, LOC101106967, LOC101104008, LOC101104268, LOC101102929, LOC101116861, LOC114118545, LOC114118325, LOC101113273 | 50.64877 | 2.36E-42 |
| MF_DIRECT | GO:0004984 | olfactory receptor activity | 42 | 1.67E-34 | LOC101118639, LOC101110175, LOC101112513, LOC101109389, LOC101109644, LOC105613588, LOC101108776, LOC101111227, LOC101106793, LOC101115825, LOC101103181, LOC101108078, LOC101107385, LOC101104270, LOC101102512, LOC101109909, LOC101106879, LOC101119160, LOC101122303, LOC101105687, LOC101122549, LOC101105947, LOC101114305, LOC101102262, LOC101104781, LOC105602384, LOC101110447, LOC101105276, LOC101107133, LOC101107550, LOC101106460, LOC101104526, LOC101106967, LOC101104008, LOC101104268, LOC101102929, LOC101122813, LOC101116861, LOC114118545, LOC101120558, LOC114118325, LOC101113273 | 11.19096 | 3.67E-33 |
| MF_DIRECT | GO:0004930 | G protein-coupled receptor activity | 43 | 8.08E-33 | LOC101118639, LOC101110175, LOC101112513, LOC101109389, LOC101109644, LOC105613588, LOC101108776, MRGPRF, LOC101111227, LOC101106793, LOC101115825, LOC101103181, LOC101108078, LOC101107385, LOC101104270, LOC101102512, LOC101109909, LOC101106879, LOC101119160, LOC101122303, LOC101105687, LOC101122549, LOC101105947, LOC101114305, LOC101102262, LOC101104781, LOC105602384, LOC101110447, LOC101105276, LOC101107133, LOC101107550, LOC101106460, LOC101104526, LOC101106967, LOC101104008, LOC101104268, LOC101102929, LOC101122813, LOC101116861, LOC114118545, LOC101120558, LOC114118325, LOC101113273 | 9.611967 | 1.18E-31 |
| MF_DIRECT | GO:0004190 | aspartic-type endopeptidase activity | 6 | 6.40E-05 | LOC101105611, LOC101108872, LOC114110083, LOC101102275, LOC101122824, LOC101122394 | 14.0958 | 7.04E-04 |
| MF_DIRECT | GO:0008483 | transaminase activity | 2 | 0.029761 | ACCSL, ACCS | 65.35325 | 0.2619 |
| INTERPRO | IPR000725 | Olfact_rcpt | 37 | 9.65E-32 | LOC101118639, LOC101110175, LOC101112513, LOC101109389, LOC101109644, LOC105613588, LOC101108776, LOC101111227, LOC101106793, LOC101103181, LOC101108078, LOC101107385, LOC101104270, LOC101102512, LOC101109909, LOC101119160, LOC101122303, LOC101105687, LOC101122549, LOC101105947, LOC101102262, LOC101104781, LOC105602384, LOC101110447, LOC101105276, LOC101107550, LOC101106460, LOC101104526, LOC101106967, LOC101104268, LOC101102929, LOC101122813, LOC101116861, LOC114118545, LOC101120558, LOC114118325, LOC101113273 | 13.02972 | 1.04E-29 |
| INTERPRO | IPR000276 | GPCR_Rhodpsn | 39 | 7.74E-31 | LOC101118639, LOC101110175, LOC101112513, LOC101109389, LOC101109644, CHRM4, LOC105613588, LOC101108776, MRGPRF, LOC101111227, LOC101106793, LOC101103181, LOC101108078, LOC101107385, LOC101104270, LOC101102512, LOC101109909, LOC101119160, LOC101122303, LOC101105687, LOC101122549, LOC101105947, LOC101102262, LOC101104781, LOC105602384, LOC101110447, LOC101105276, LOC101107550, LOC101106460, LOC101104526, LOC101106967, LOC101104268, LOC101102929, LOC101122813, LOC101116861, LOC114118545, LOC101120558, LOC114118325, LOC101113273 | 10.85779 | 4.18E-29 |
| INTERPRO | IPR017452 | GPCR_Rhodpsn_7TM | 39 | 1.06E-29 | LOC101118639, LOC101110175, LOC101112513, LOC101109389, LOC101109644, CHRM4, LOC105613588, LOC101108776, MRGPRF, LOC101111227, LOC101106793, LOC101103181, LOC101108078, LOC101107385, LOC101104270, LOC101102512, LOC101109909, LOC101119160, LOC101122303, LOC101105687, LOC101122549, LOC101105947, LOC101102262, LOC101104781, LOC105602384, LOC101110447, LOC101105276, LOC101107550, LOC101106460, LOC101104526, LOC101106967, LOC101104268, LOC101102929, LOC101122813, LOC101116861, LOC114118545, LOC101120558, LOC114118325, LOC101113273 | 10.11012 | 3.81E-28 |
| INTERPRO | IPR050427 | Olfactory_Receptors | 8 | 4.53E-08 | LOC101104526, LOC101122303, LOC105613588, LOC101122549, LOC101122813, LOC101105276, LOC101104781, LOC101104270 | 23.63349 | 1.22E-06 |
| INTERPRO | IPR033121 | PEPTIDASE_A1 | 4 | 8.95E-04 | LOC101105611, LOC101102275, LOC101122824, LOC101122394 | 21.00754 | 0.016103 |
| INTERPRO | IPR001461 | Aspartic_peptidase_A1 | 4 | 8.95E-04 | LOC101105611, LOC101102275, LOC101122824, LOC101122394 | 21.00754 | 0.016103 |
| INTERPRO | IPR021109 | Peptidase_aspartic_dom_sf | 4 | 0.002447 | LOC101105611, LOC101102275, LOC101122824, LOC101122394 | 14.79662 | 0.03776 |
| INTERPRO | IPR012848 | Aspartic_peptidase_N | 3 | 0.006764 | LOC101102275, LOC101122824, LOC101122394 | 24.0794 | 0.091313 |
| INTERPRO | IPR050478 | Ethylene_sulfur-biosynth | 2 | 0.009241 | ACCSL, ACCS | 212.7014 | 0.110886 |
| INTERPRO | IPR004839 | Aminotransferase_I/II_large | 2 | 0.058578 | ACCSL, ACCS | 32.72329 | 0.632648 |
| KEGG_PATHWAY | oas04740 | Olfactory transduction | 40 | 7.46E-28 | LOC101118639, LOC101110175, LOC101112513, LOC101109389, LOC101109644, LOC105613588, LOC101108776, LOC101111227, LOC101106793, LOC101115825, LOC101103181, LOC101108078, LOC101107385, LOC101104270, LOC101102512, LOC101109909, LOC101106879, LOC101119160, LOC101122303, LOC101105687, LOC101122549, LOC101105947, LOC101114305, LOC101102262, LOC101104781, LOC101110447, LOC101105276, LOC101107133, LOC101107550, LOC101106460, LOC101104526, LOC101106967, LOC101104008, LOC101104268, LOC101102929, LOC101122813, LOC101116861, LOC114118545, LOC114118325, LOC101113273 | 7.498101 | 2.01E-26 |
| KEGG_PATHWAY | oas04974 | Protein digestion and absorption | 5 | 0.005238 | LOC101105611, LOC101108872, LOC101102275, LOC101122824, LOC101122394 | 6.997758 | 0.070716 |
